# Toad zoonyms mirror the linguistic and demographic history of Greece

**DOI:** 10.1371/journal.pone.0283136

**Published:** 2023-03-29

**Authors:** Leonidas-Romanos Davranoglou, Leonidas Embirikos

**Affiliations:** Oxford University Museum of Natural History, University of Oxford, Oxford, United Kingdom; Chulalongkorn University, THAILAND

## Abstract

The common toad (*Bufo bufo*) has been the subject of many folk tales and superstitions in Western Europe, and as a result, it is characterised by numerous common names (zoonyms). However, the zoonyms of the toad and its associated traditions have remained unexplored in the Balkans, one of Europe’s linguistic hotspots. In the present study, it was attempted to fill this knowledge gap by focusing on Greece, where more than 7.700 individuals were interviewed both in the field and through online platforms, in order to document toad zoonyms from all varieties and dialects of Greek, as well as local non-Greek languages such as Arvanitika, South Slavic dialects, and Vlach. It was found that the academically unattested zoonyms of the toad provide an unmatched and previously unexplored linguistic and ethnographic tool, as they reflect the linguistic, demographic, and historical processes that shaped modern Greece. This is particularly pertinent in the 21^st^ century, when a majority of the country’s dialects and languages are in danger of imminent extinction–and some have already gone silent. Overall, the present study shows the significance of recording zoonyms of indigenous and threatened languages as excellent linguistic and ethnographic tools that safeguard our planet’s ethnolinguistic diversity and enhance our understanding on how pre-industrial communities interacted with their local fauna. Furthermore, in contrast to all other European countries, which only possess one or only a few zoonyms for the toad, the Greek world boasts an unmatched 37 zoonyms, which attest to its role as a linguistic hotspot.

## Introduction

When two distinct populations come into extensive contact, their relations are often characterised by inequalities. The group that is superior technologically or economically usually prevails over the weaker one in various aspects of their common life. This unequal relationship affects language as well, where the language of the weaker group is displaced or replaced by that of the dominant one. This phenomenon is described linguistically, where the **substrate** denotes the vestiges of a language of low prestige relative to the **superstrate**, i.e. the typically high prestige idiom that displaces it [1, 2]. However, even when the substrate language goes extinct, some of its vocabulary may pass into the dominant language that replaced it, serving as a reminder of the prior linguistic situation of that particular population or area. Substrate terms, therefore, act as “linguistic fossils”, and are useful tools for understanding linguistic, demographic, and historical processes [2]. This is particularly true for understanding phenomena that took place in prehistoric times and periods neglected by historiography, when often no other source of information is available. Some indicative examples are provided here: having a Balkan background in mind, substrate terms in ancient Greek, such as the toponym Parnassus and the word labyrinth, were used to elucidate the origin of the pre-Greek population, and revealed putative links with the Luwian culture of Asia Minor [3]. On the other hand, the absence of pre-Greek substrate terms in the Pindus mountains highlighted Epirus as a possible cradle of the proto-Greeks [4]. The evidence for a relationship between the elusive Albanian language and its allegedly Latinised sisters, Aromanian (referred hereafter as Vlach, its exonym) and Romanian, is mainly based on shared substrate terms that are found only in these languages [5–12]. Substrate place names are also used to identify archaeologically “invisible” populations, such as Slavs and their massive migrations to the Balkan peninsula from the 6^th^ century onwards [13–17].

Zoonyms and phytonyms form the basis for many substrate terms, especially when they describe organisms of low economic value, which often escape the attention of the stratum (i.e. the dominant language). The English term *brock* (= badger), for example, derives from the Proto-Celtic term *brokkos and is a testament of Britain’s Brythonic past prior to the Anglo-Saxon invasions [18]. The survivability of a given zoonym is enhanced when the animal in question is accompanied by some kind of chthonic beliefs, which must be concealed from conquering elites, or when it possesses pharmacological properties that are known only by a select group of people, such as healers [19].

A group of animals of significant pharmacological and anthropological importance are the true toads, that is, the amphibians of the family Bufonidae. All bufonids bear a pair of large parotid glands on the back of their heads (as well as many smaller glands along their bodies), which secrete a plethora of toxic substances known as bufotoxins (e.g. bufogenin, bufotenin) when the animals are disturbed or threatened [20, 21]. Ingestion of these toxins causes unpleasant symptoms to prospective predators (vomiting, cardiomyalgia, dizziness, reduced vision, and difficulty breathing) [22, 23], and low amounts of the secretions found in certain toad species cause psychedelic hallucinations in humans [23–25]. This defensive property of toads has caused them to fall victims of a multitude of superstitions and beliefs, a subset of which are reported below.

In Central and Western Europe, the European common toad (*Bufo bufo*) is widely regarded as a “lowly” species, as it has been associated with witchcraft, delinquent, and vile behaviours for centuries, if not millennia. The toad has been regarded throughout time as a bad omen associated with the underworld. For instance, in the Middle Ages, three toads adorned the devil’s coat of arms [26, 27]. Also, ordinary people considered toads to be dangerous animals that could spit fire and poison. During the witch-hunting frenzy of the Middle Ages, many believed that the toad was the trusted advisor or family relative of these women (*witch’s familiar*), the animal being cared for and dressed by them, as it was essential for the success of their spells [27]. These beliefs stem not only from the toads’ ominous–for some–appearance, but also from the hallucinogenic secretions of their glands, which linked them to magical practices and especially to the witches’ alleged flying capacity. During rituals that could be described as shamanistic, the “witches” would smear the toad secretions on their groin and armpits, which created a sensation of flying. These beliefs took a definitive form by the Spanish Inquisition, which codified the toad’s links with witchcraft and what was considered as aberrant sexuality by the Church, particularly in the famous Logroño Trials (1610–1614), history’s largest witch hunt [28]. This took place in the Basque Country and resulted in the arrest of over 7.000 people, mostly women [28]. Of course, the so-called witches were ordinary rural women who retained the collective wisdom and ancestral knowledge of their country’s recent pagan past [29].

Due to the multitude of superstitions that characterise the common toad, and the survival of substrate zoonyms that describe this species in several European languages (as, for instance, in Spain, where it is known as *sapo*, instead of the Latin *bufo*; [30]), the potential utility of *Bufo bufo* in linguistics and anthropology merits further investigation. The present study addresses the latter point.

Coming from different scientific backgrounds (Davranoglou, L.-R., biologist-zoologist, Embirikos, L., historian) the authors’ research in the Greek countryside and their interactions with the locals, led both of them to independently realise that this country is characterised by an unusually large number of zoonyms for the common toad [31], unlike other European languages, where only few terms are used to describe this animal. Furthermore, the authors were unable to locate many of the toad zoonyms that they encountered in the field in either the domestic or in the international linguistic literature. Other than Lithoxou, who recorded some of the toad’s zoonyms and their distribution [32], the linguistic and anthropological value of this species had remained unexplored.

At an early stage [31], the authors realised that, in Greece, certain toad zoonyms were geographically restricted, but were also considerably more diverse than those of any other animal in Greece. Their distribution probably indicated the existence of dialectal regionalism that needed to be explored further. Also, unlike Western Europe, where this animal is of great anthropological value, the beliefs of the Balkan peoples regarding the toad have not been well documented, and this is especially true for Greece. This situation is particularly paradoxical, as in discussions with farmers and herders throughout mainland Greece and the Balkans in general, the authors discovered an extremely widespread (but scientifically unrecorded) belief that the toad attacks goats, sheep, and cattle, in order to suckle their udders, with seemingly lethal consequences for the animals. The above reasons made it obvious that the zoonyms of this species and associated traditions merited further study.

Unlike neighbouring Balkan countries, where linguistic maps are readily available [33–37], the dialectal varieties of modern Greek (especially those of the mainland), although well-studied at the regional level, have rarely been researched under the lens of comparative historical linguistics, nor have they been mapped [38]. As a result, the boundaries of Greek dialectal varieties, their phylogenetic relationships, their contact with different idiomatic forms of Greek or other languages, as well as the historical processes that shaped their evolution and distribution, have often remained elusive [38]. Moreover, the distribution of non-Greek languages in Greece, as well as their influence on Greek dialectal varieties has often been obscured in the public sphere, where they have been downgraded to mere dialects or idioms of unspecified languages [39–42].

Having the above context in mind, the primary goal of this study is to document the diversity, distribution, and origin of toad zoonyms of Greece, and to explore their possible linguistic, historical, demographic, and anthropological value.

The present study will demonstrate that, due to their frequent role as substrate terms, toad zoonyms constitute an excellent–and so far untapped–resource on the linguistic and demographic history of Greece, especially for poorly-attested historical eras such as the 6^th^ century AD, when Slavs massively settled the Balkan peninsula. Importantly, they further elucidate how the mixing, levelling, and simplification of different varieties and dialects of Greek (**koineization**) led to the present state of Greece’s linguistic landscape, which ultimately also shaped Standard Modern Greek. Finally, this work contributes to the preservation of the folkloric wealth of Greece, by recording beliefs, superstitions and zoonyms that are absent from the anthropological literature of the country.

## Material and methods

### 1. Recording of the zoonyms of the common European toad

The study recorded the zoonyms of the common European toad both from the field and through social media platforms over a span of 35 years, covering almost the entirety of surviving Greek idioms and dialectal varieties, as well as the country’s non-Greek languages [Arvanitika (Albanian dialects), Turkish (Gagauz), Slavic (Macedonian, Bulgarian, Pomak), and Vlach (Aromanian, Meglenoromanian)]. Data collected online are based on more than 4.700 informants who volunteered to participate in this study through social media groups (Facebook). Together with the field data, the total observations for this research exceed 7.700 informants, rendering this work as one of the most comprehensive zoonymic studies in Greece. Written and verbal consent was obtained from all participants (Methodology in S1 Text) and all online data collection was undertaken with the permission of the Social Sciences and Humanities Interdivisional Research Ethics Committee (SSH IDREC) of the University of Oxford (permit number R81032/RE001). Sampling locations and the employed methodology are summarized in S1 Text.

**Fig 1:**
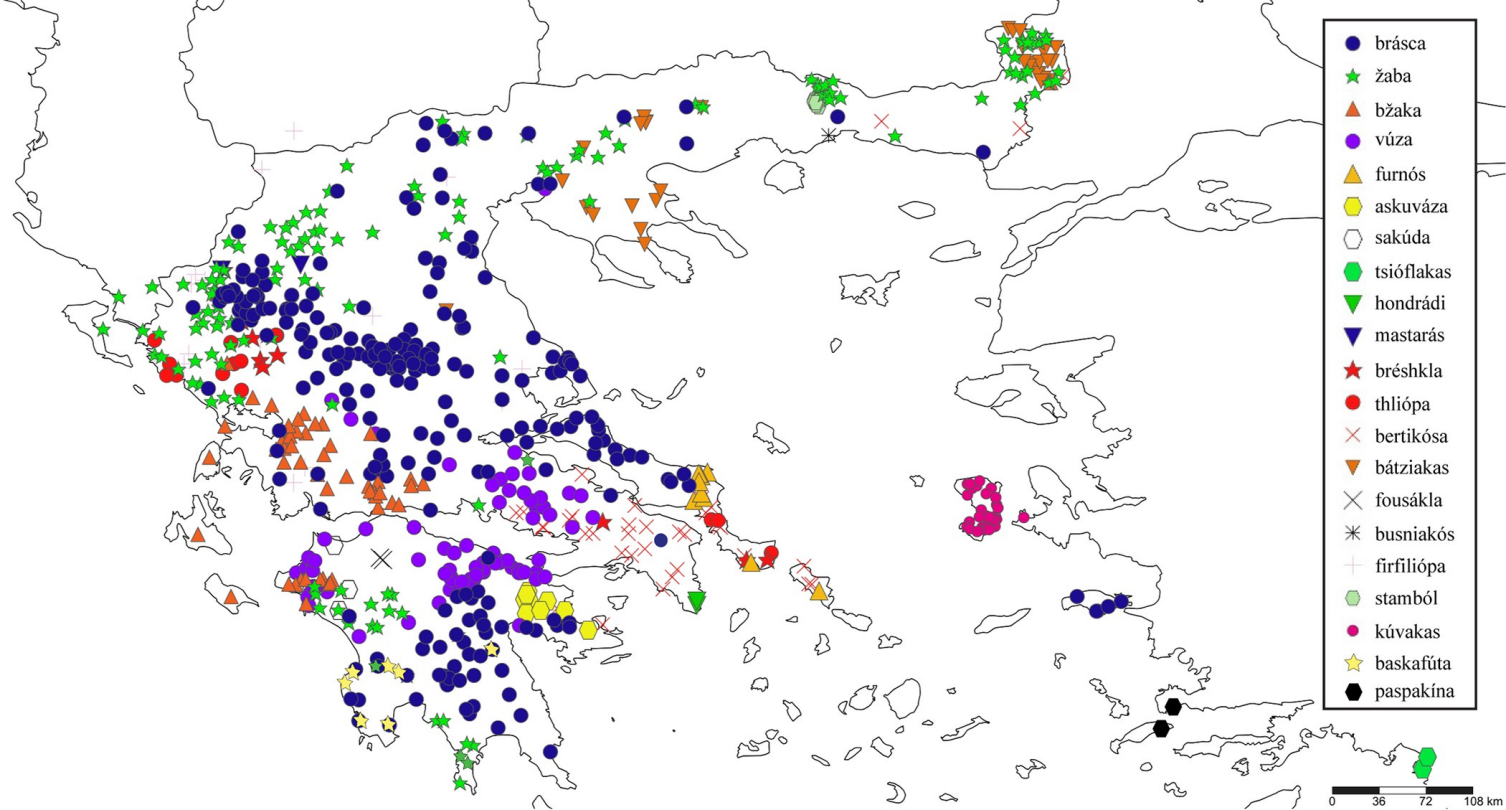
Sampling locations where the main zoonyms of the common toad were recorded, both in the field and through social media. The number of sites does not equal the total number of informants, as multiple individuals were interviewed from each locality. Zoonyms of limited geographic range are not shown.

**Fig 2:**
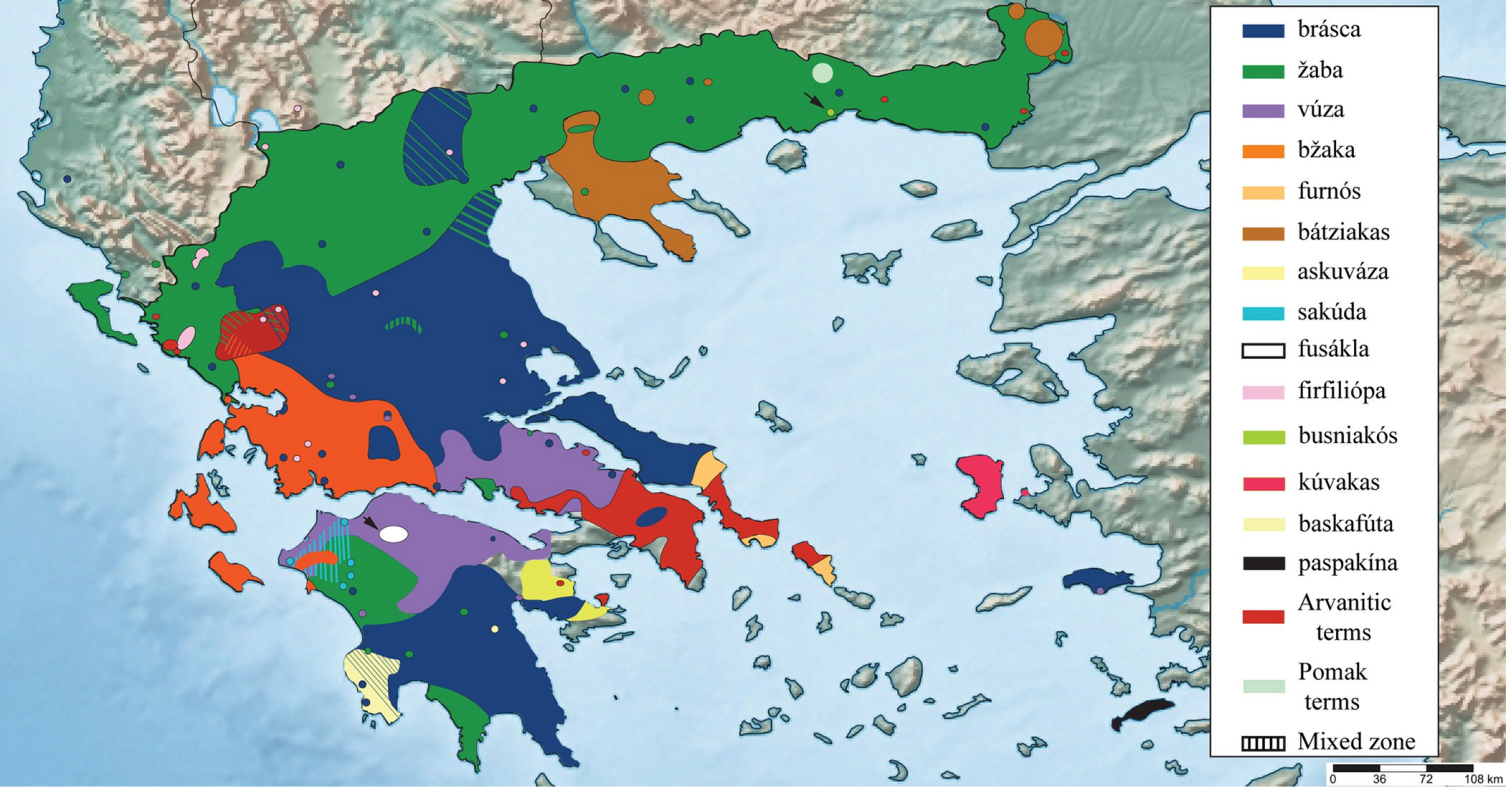
The geographic distribution of the main zoonyms of *Bufo bufo* in Greece, based on the data of Fig 1. Zoonyms of limited geographical distribution are not depicted. Dashed colours indicate the existence of mixed zones where multiple zoonyms co-occur. The unicoloured areas are schematic and do not exclude the existence of linguistic “islands” of different zoonyms within their boundaries, which may have not been identified in the present survey. Also, in mixed zones where multiple zoonyms co-occur, the degree of use of each term by the local population (i.e whether one zoonym is dominant, while another is falling out of use) is not depicted. It should be stressed that the maps of Central and Eastern Macedonia, as well as those of Thrace, do not depict the drastic demographic and linguistic changes that took place in these regions during the 20^th^ century. Uncoloured areas denote parts of Greece that were not sampled by the present study, urban centres, and regions where the common toad does not occur.

The sum of this study’s observations is presented in Figs 1 and 2. For broadly used zoonyms, their distribution and variants are summarised in the Supplementary Information (S1 Table. Geographical distribution of zoonyms at village level).

### 2. Statistical analysis

Graphs were created using the statistical program R version 4.1.0 [43] and ggplot2 [44]. In addition, a Kruskal-Wallis test was performed, in order to examine the relationship between altitude and zoonym used, using the kruskal.test function in R. The datapoints used are provided in S1 Table.

### 3. Maps and figures

The maps were constructed using the SimpleMappr program [45], based on the geographic coordinates of each site examined. The drawings were made using Adobe Illustrator CS6.

### 4. Resources of ancient Greek inscriptions and names

To study the distribution of the ancient Greek term *φρῦνος* (phrýnos = common toad) in classical and Roman antiquity, apart from a literature search, online catalogues of ancient inscriptions and names [46, 47] were consulted, covering the period between 600 BC and AD 200. Inscriptions from religious centers such as Delphi, Epidaurus, and Olympia, which attracted people from all over the Greek world, were generally not included in this study.

### 5. Employed terminology

Throughout the text, the Greek-speaking populations of Medieval to modern times are referred to as Romans, which may be surprising to an international readership. However, the emic view of the Greek-speaking residents of the Eastern Roman Empire, as well as the Latin states and the Ottoman Empire that succeeded it, was that they were Romans (Ρωμαίοι-Ρωμιοί), and that the language they spoke was Roman (Ρωμαίικα = Romeika) [48–50]. From early Medieval times, a key element of “Romanness” was Greek speech, as it became the main, and eventually the official language of the Eastern part of the Roman Empire. Moreover, Christian Greek-speakers usually differentiated themselves from neighbouring “others” of the same faith, such as speakers of Albanian, Vlach, Slavic [49, 50]. It should be noted that although the emic term Romeyka is used to refer to the Pontic variety currently spoken by Muslim Turks in the Trabzon area, following the terminology of [51], historically, most speakers of both mainland and Asia Minor Greek-speaking areas would also describe their own language as /roméika/. The term Roméika is still being used by almost the entirety of indigenous Greek-speaking populations outside Greece, with the exception of those of southern Italy.

When transcribing Greek placenames into the Latin alphabet, the official transcription of the Hellenic Republic was used. For the transcription of ancient Greek personal names and city-states, the system employed in classical studies (e.g. where *φ* is transcribed to *ph*, instead of *f* for modern Greek) was followed.

Finally, with the exception of Muslim Greek-speakers living in the Trabzon area, all data from Turkey refer to Greek-speakers from the Ottoman Empire and the succeeding Republic of Turkey, who were resettled in Greece from 1922 to 1925.

### 6. Use of genetic data

Recent years have witnessed the beginning of a revolution in population genetics, which is now a key tool for studying the ultimate origins of humanity [e.g. 52, 53]. Examining the genome of modern populations and ancient remains (archaeogenetics) has also proven to be of great importance for linguistics, as it sheds light on the origin and transmission of various languages, such as the dispersals of the Indo-Europeans from the Caspian steppes to the rest of Europe and South Asia [54, 55], the Bantu expansion [56], the transmission of Uralic languages into Asia and Europe [57, 58], and the entry of Celtic speech into Britain [59]. In a Balkan context, several studies have examined the introduction of Greek into Greece [60], the possible relationship between the Daunians of Italy to populations from the West Balkan Iron Age [61], the migrations of the Arbëreshë to Italy [62], and the ancestry contribution of Slavs to the present-day peoples of the Balkan peninsula [63, 64].

In the present study data from population genetics are occasionally used as a complementary resource for understanding migrations within and beyond the Greek region and the Balkans in general. Although genetic data do not provide conclusive evidence for the dispersal of a particular language or a given zoonym, they can be useful for understanding the linguistic and demographic processes that took place in periods that have been inadequately recorded historically. It should be stressed that the genetic data consulted herein should not be treated as absolute proof, but as a complementary resource for understanding and interpreting archaeological, linguistic, and historical data.

## Results 1: The dominant zoonyms of *Bufo bufo* in Greece

### 1. *Φουρνός* (*furnós*) and related terms

**Φουρνία (furnía), σκαφουρνία (skafurnía).** South Euboea, region of Kymi. **Φουρνιά (furniá).** Southern Euboea, region of Aliveri and Karystos. **Φουρνός (furnós)**. Central and southern Andros, Karystos. **Φουρνόν (furnón)**. Turkey, Black Sea coast, Greek-speaking regions of Trabzon province.

For detailed distribution and additional derivatives, refer to S1 Text (chapter 2.1.1.).

*Discussion*. The zoonym *φουρνός* (*furnós*) and its relatives result from metathesis of the perirrhotic vowel of the term *φρῦνος* (*phrýnos* = toad) or *φροῦνος* (*phrúnos*) of ancient Greek. The likeliest etymology of the term stems from proto-Indo-European *bʰerH-‎ (= brown), although Beekes [65] does not exclude the possibility of a pre-Greek origin of unknown etymology. Regarding the modern descendants of this term, the asynizesised form *φουρνία* (*furnía*) is found exclusively in the area of Kymi in Euboea Island (S1 Text, chapter 2.1.1.), and the variant *σκαφουρνία* (*skafurnía*, from Greek *σκάπτω* (= to dig) + *φουρνία* = *digging furnía*) describes the tendency of the common toad to bury itself into the ground. In those areas where the Euboean dialect is synezised, the term becomes *φουρνιά* (S1 Text, chapter 2.1.1.). The feminisation of this zoonym is a secondary development, as the ancestral forms *φρῦνος* and *φροῦνος* are masculine, and their gender is maintained in the Cycladic idiom of Andros (*φουρνός*) and in Pontic (*φουρνόν*).

**Fig 3:**
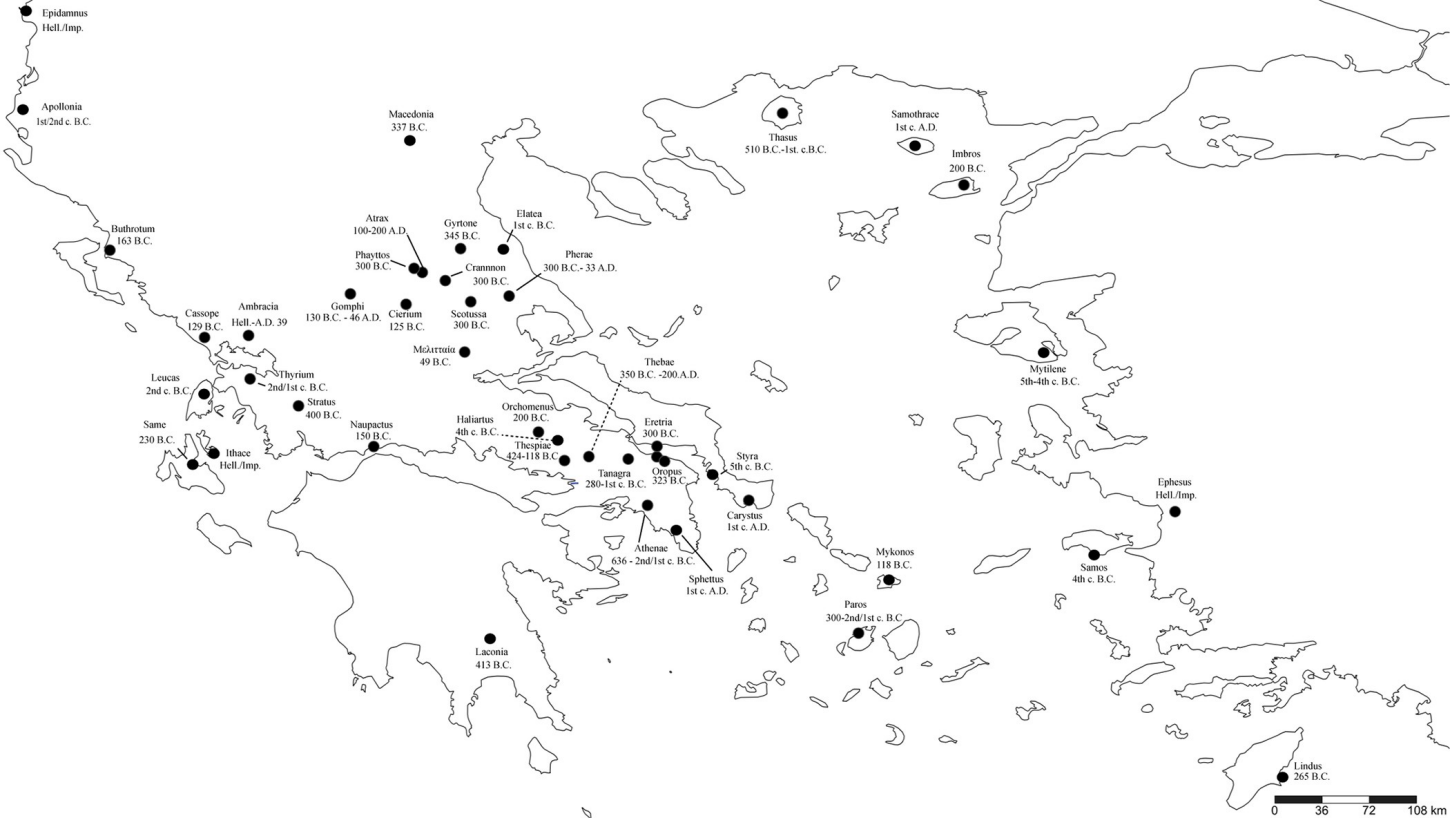
The distribution of personal names derived from *φρῦν*- and *φροῦν* in the Greek-speaking world between 600 BC and AD 200.

**Fig 4:**
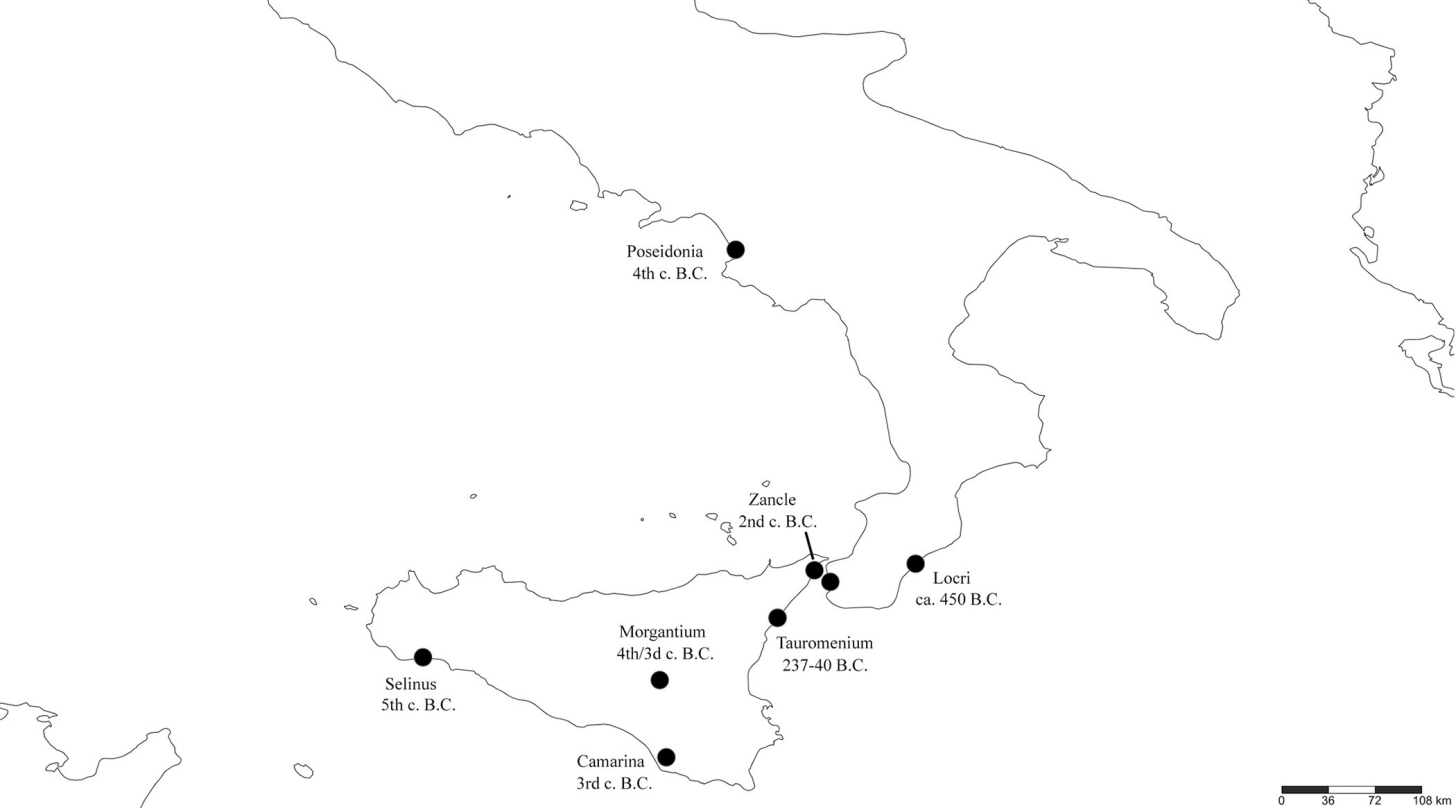
The distribution of personal names derived from *φρῦν*- and *φροῦν*- in Magna Graecia between 500–200 BC.

The zoonym *φρῦνος* forms the base of many ancient Greek personal names, such as Φρῦνος, Φρύνη, Φρυνοκλῆς, Φρυναῖος, Φρυνίας, Φρύνιχος, Φρυνίδης, Φρυνώνδας, etc., which are attested in a large number of inscriptions [66–71]. It is evident from Fig 3, that in classical antiquity, personal names derived from *φρῦνος* and *φροῦνος* were found primarily in Attica, Boeotia, Thessaly, and their colonies, i.e. they have a clear Aeolic-Attic-Ionic distribution. Indeed, a previous study [69] confirms that the bases *φρῦν*- and *φροῦν*- represent common onomastics in Boeotia from 700–200 BC (although they are erroneously attributed to the frog, and not the common toad). The few records of these onomastics from Western Greece, the colonies of Apollonia and Buthrotum, are dated in their majority to the Hellenistic and Imperial Roman periods (Fig 3), and likely represent an influence from the Hellenistic koine, which was largely based on the Attic-Ionic dialect. The presence of this term in Macedonia (Fig 3) may also be a consequence of the Atticisation of the region [72]. The situation in Magna Graecia is more complex, as onomastics based on *φρῦν*- and *φροῦν*- are found in both Doric (e.g. Selinus, Poseidonia), and Ionic colonies (Tauromenium, Zancle) (Fig 4). Moreover, it is not known whether the term *φρῦνος* was used as a zoonym in Magna Graecia, or solely as an onomastic.

**Fig 5:**
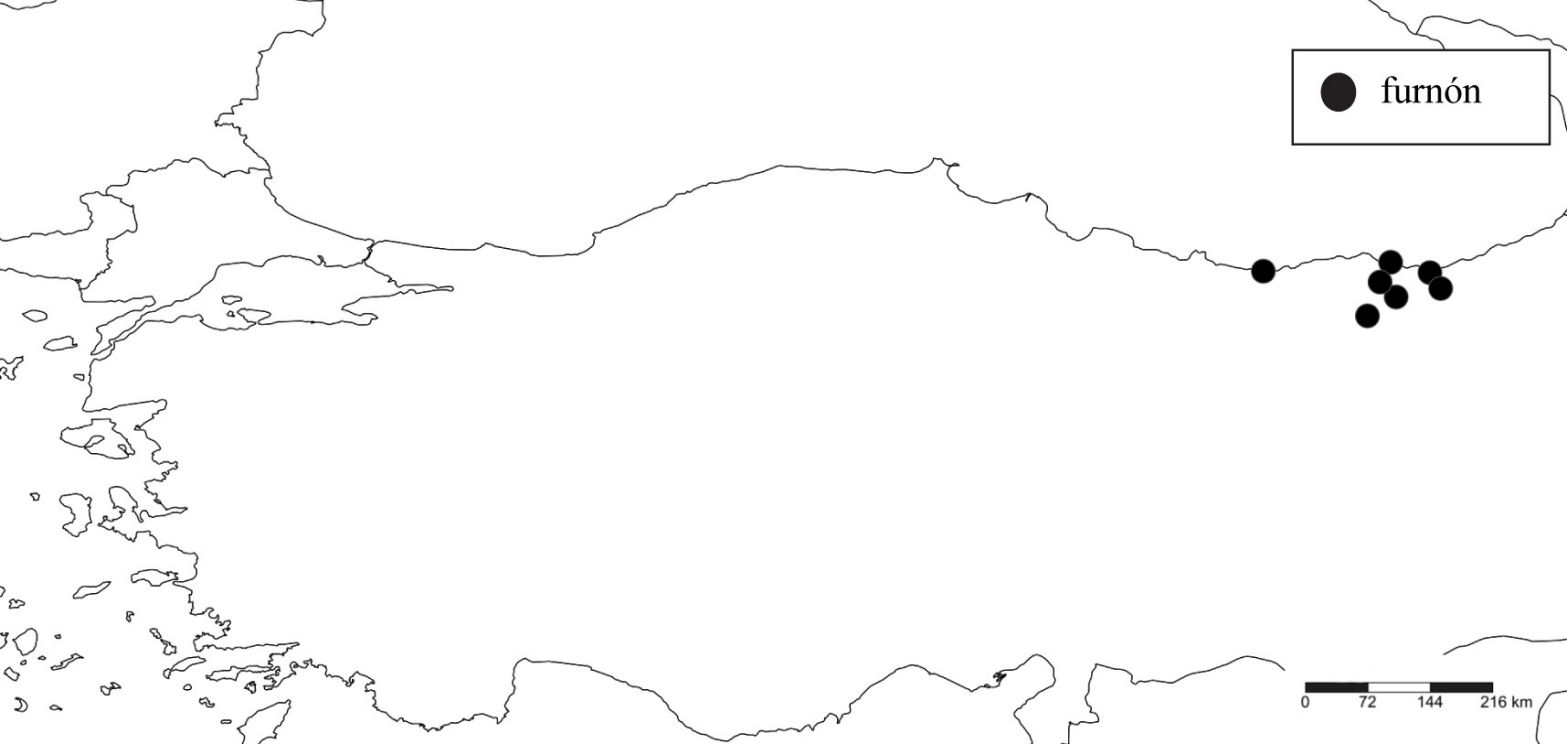
The distribution of the term *φουρνόν* (*furnón*) in Pontus in the early 20^th^ century, based on the origin of the Pontian refugees who still use this zoonym, but also on the current speakers of Romeyka in Turkey.

Although no records of the use of the term *φρῦνος* after AD 200 could be found (either in inscriptions or in the literature), based on the current distribution of its descendant zoonyms, it is suggested that it may have survived as a possible substrate term of Ionian speech in Euboea and the Black Sea (and potentially Andros) and throughout the integral parts of what constituted the ancient Ionic world. Indeed, the discovery of the inscription *Φρύνη Σινωπίς* (Phryne from Sinope in Pontus) in Attica (*IG* II^2^ 10361 = FRA 6880 [47]), which is dated to the Hellenistic era, suggests that this zoonym was used in personal names in the region of Pontus at least since then, if not much earlier. The survival of descendants of the ancient Greek term *φρῦνος* only on islands (Andros, Euboea) (Fig 2) and linguistic islets of Greek speech (Pontus) (Fig 5), deems probable that they were replaced in the wider Greek-speaking world by different zoonyms of the prevailing common speech (koine) of the Middle Ages (early and late), but also of later periods.

**Fig 6:**
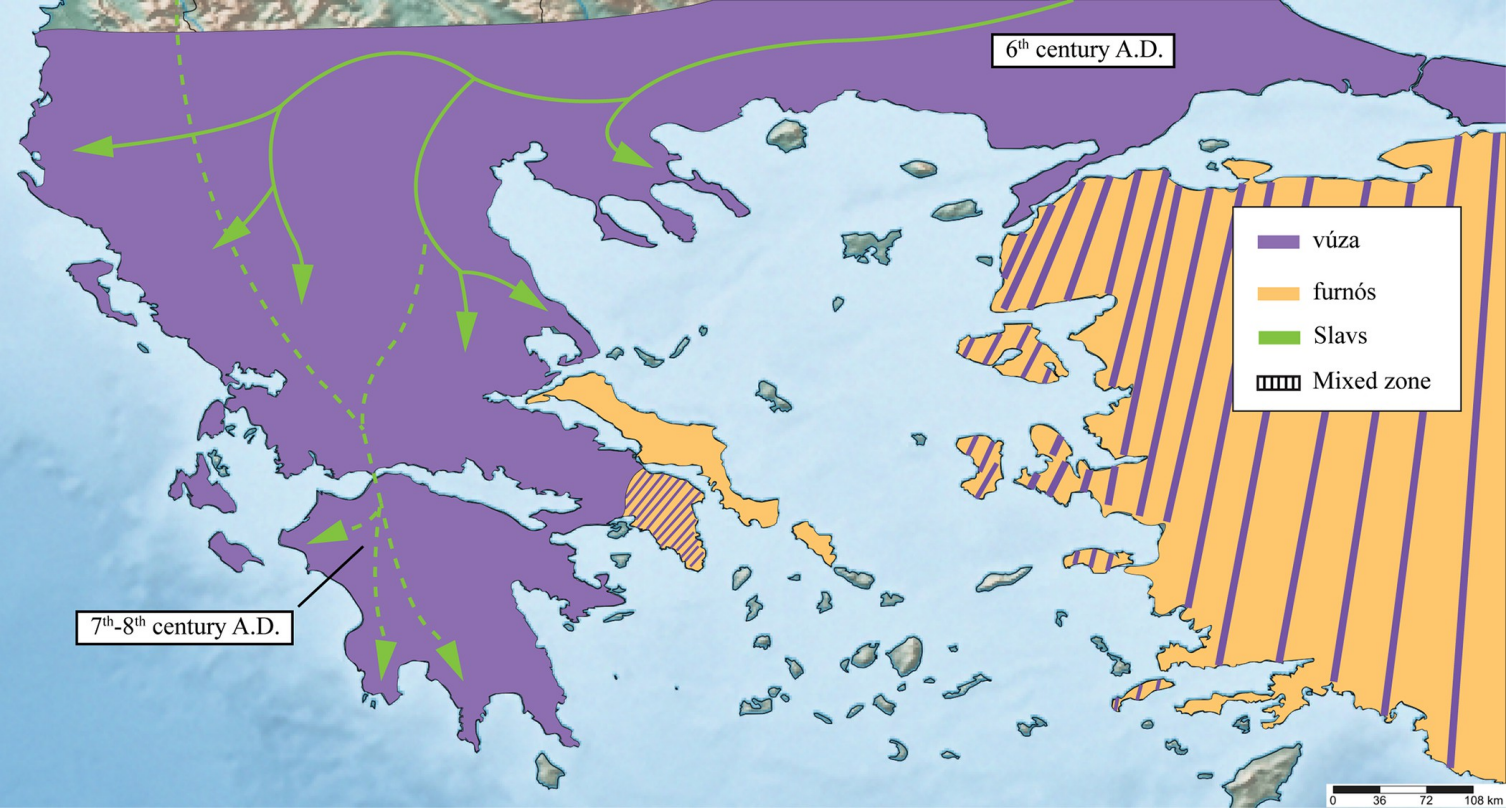
Hypothetical distribution of the zoonyms *βούζα* (*vúza*) and *φουρνός* (*furnós*) in the 6^th^ century AD, immediately prior to the Slavic migrations. The green arrows represent the waves of Slavic settlement according to [13, 77, 78]. The green dotted line indicates the uncertainty about the homeland of the Slavic populations that invaded the Peloponnese. The mixed orange-purple zones in Attica and Asia Minor are speculative, as the zoonyms used in those areas is not known.

**Fig 7:**
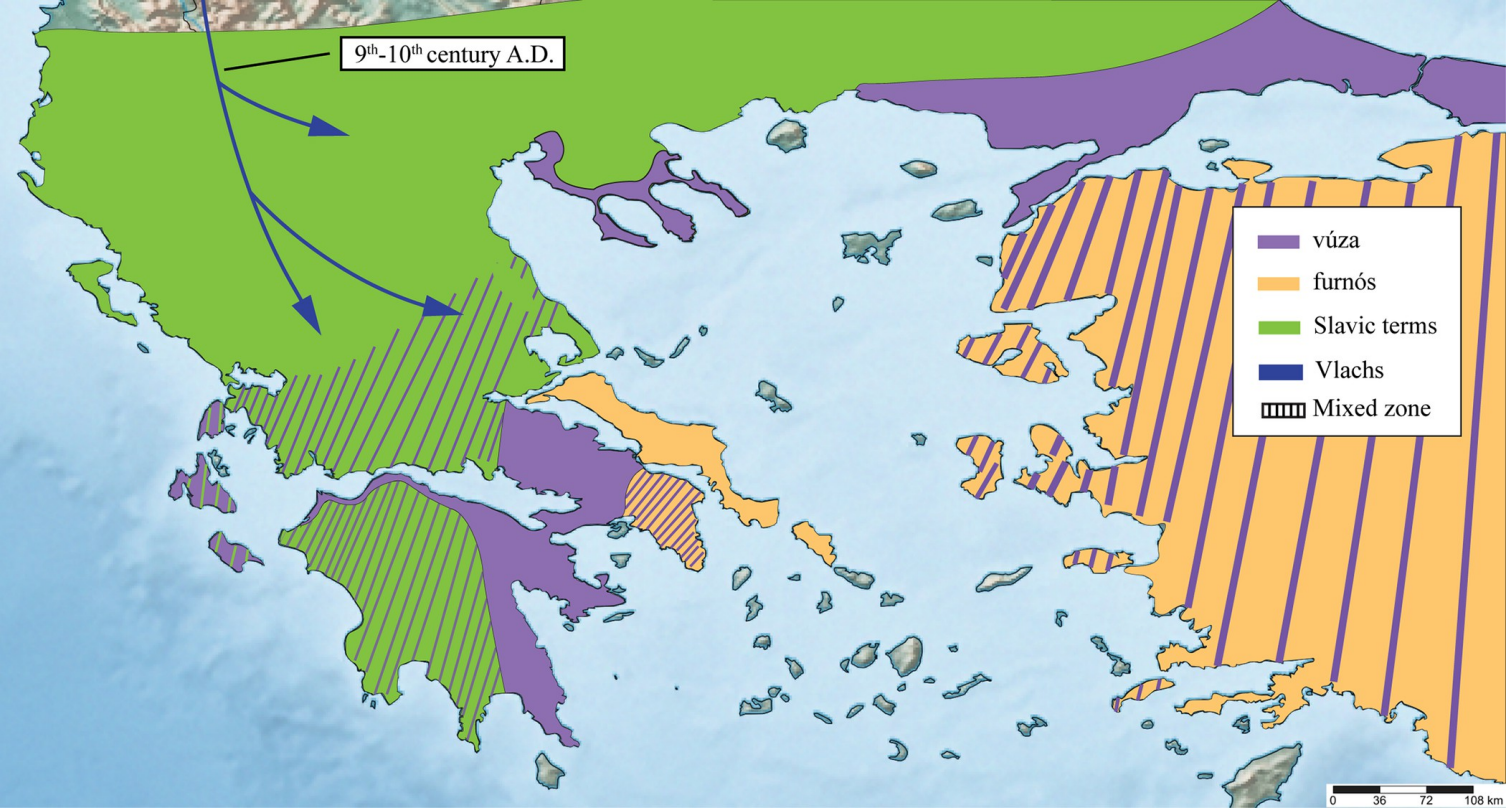
Hypothetical distribution of toad zoonyms in Greece shortly after the Slavic settlements (7^th^–9^th^ century AD), during the first Vlach movements in the area (blue arrows). The spread of each zoonym was designed according to data from its current distribution, as well as contemporary historical sources. In particular, the distribution of Slavic terms was plotted based on evidence of strong Slavic presence, such as Slavic place names and sclaveniae (i.e. areas of dense Slavic settlement) [14]. The hypothetical presence of the term *βούζα* (*vúza*) in the Peloponnese, Boeotia, and Macedonia was also based on its current range and the areas known to have remained under Roman Imperial control throughout the Migration Period [48, 49, 78]. It should be noted that this map does not exclude the likely presence of large Greek-speaking populations within areas where Slavic terms predominated, who continued using Greek zoonyms, until they were gradually replaced by Vlach ones.

**Fig 8:**
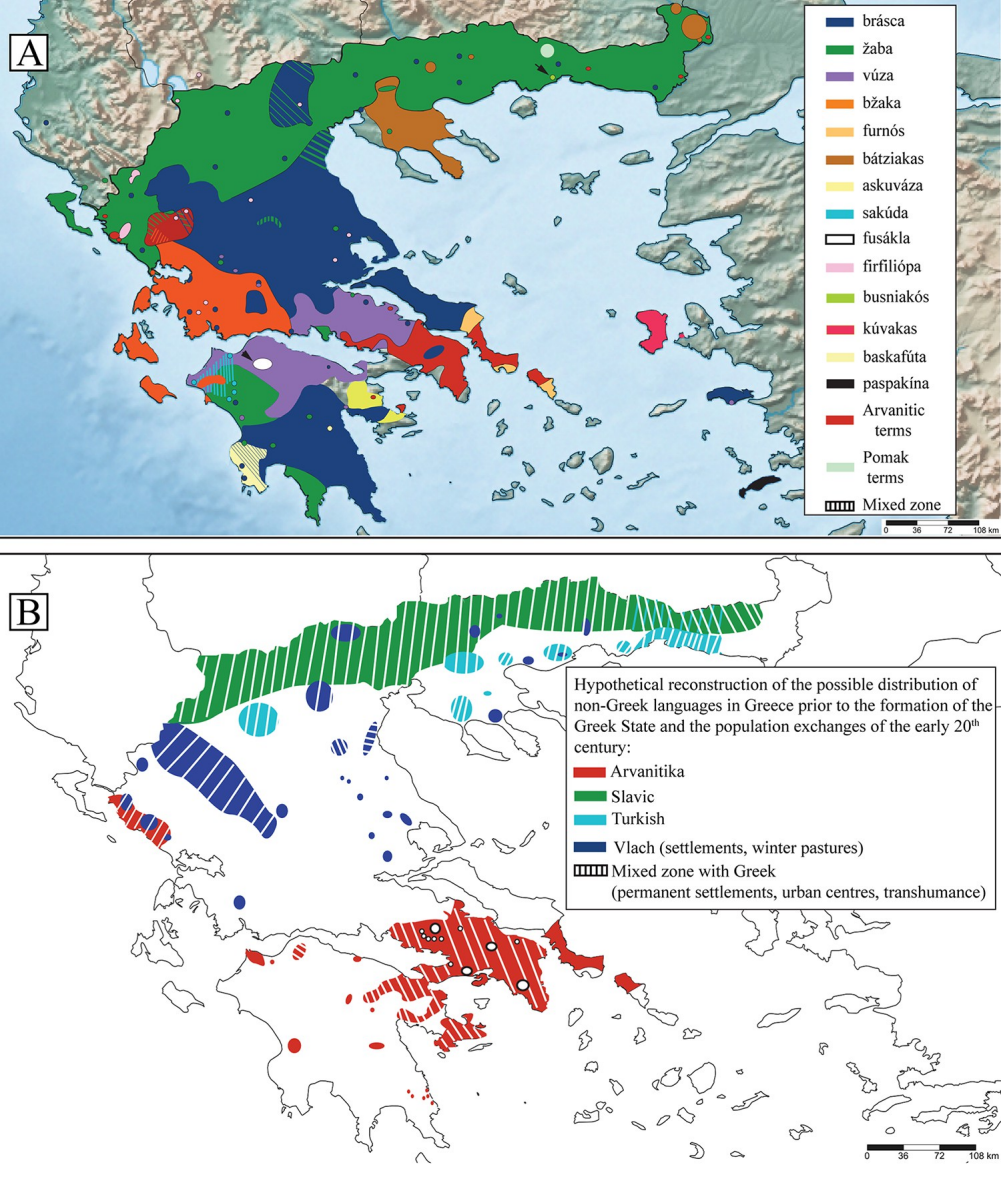
(Α) The current distribution of toad zoonyms, in relation to (B) the distribution of the main non-Greek languages of Greece in the early years of the 20^th^ century, according to [41, 79–81], and the maps according to [82, 83]. Coloured areas indicate only the dominant (but not unique) language of the rural population within their boundaries, and not the inhabitants’ ethnic identity or religion. Also, in the 20^th^ century, most of these areas were bilingual. The coloured areas include zones of movement of transhumant pastoralists, and do not necessarily indicate permanent settlement, nor do they indicate population density, which was higher in the (mainly) Greek-speaking towns. For Vlachs in particular, many of the coloured areas (e.g. in Thessaly) functioned only as winter pastures. It should be emphasised that the map depicted here is only indicative of the situation in the beginning of the 20^th^ century and not of the present one, as the linguistic and demographic composition of the region changed radically with the Balkan Wars and the population exchanges determined by the Treaties of 1913, 1914, 1919 (Neuilly) and 1923 (Lausanne) [84–87]. It should also be noted that the dense Greek-speaking populations of eastern Thrace, western-northeastern Asia Minor, and southern Albania are not depicted, as this map only concerns the linguistic situation within the borders of the modern Greek State.

**Fig 9:**
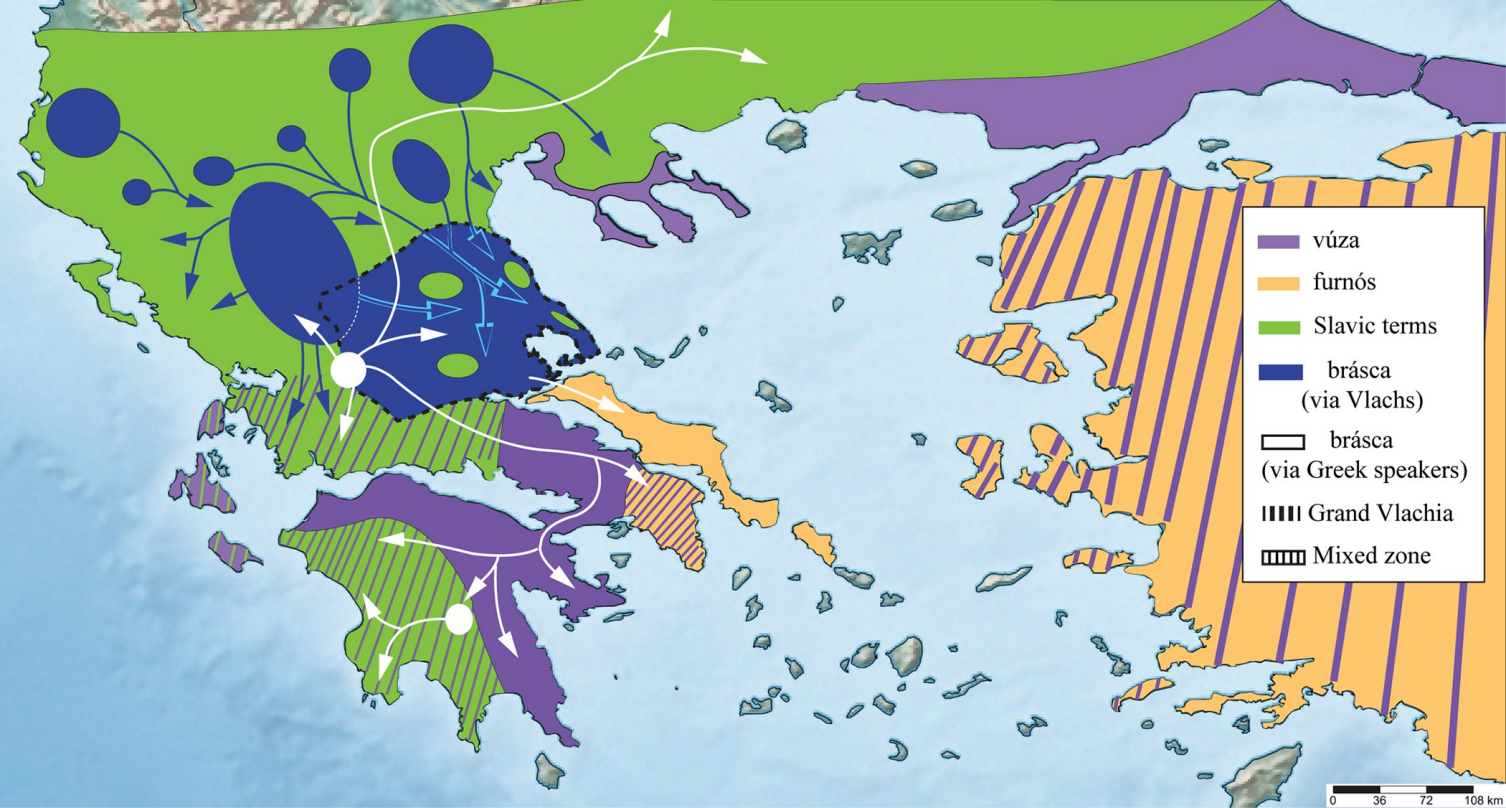
Hypothetical transmission of toad zoonyms postdating the Vlach settlements (9th century onwards). The arrows show the transmission of the term *μπράσκα* (*brásca*) by Vlachs (blue) and by Greek speakers (white) respectively, in an indeterminate time frame. The bold dash lines indicate the approximate borders of Grand Vlachia, according to [88, 89]. The distribution of all zoonyms is based on their current range, while the blue circles that act as sources of the term *μπράσκα* represent the recorded permanent settlements of the Vlachs and the annual transhumance routes that they followed [42, 79, 90, 91].

The current distribution of the terms *φουρνός*-*φουρνία*-*φουρνιά* in Kymi, Aliveri, Karystos, and throughout central and southern Andros (Fig 2), indicate that they might have been the dominant zoonyms at least in Central and South Euboea and the entirety of Andros (Figs 6, 7 and 9) prior to the 15^th^ century mass migrations of Arvanites to parts of these islands (Fig 8). Indeed, the archaic dialectal varieties of Kymi, Old Athens, Aegina, and the Cycladic dialectal variety of Andros (now only found in the valley of Chora), might have formed a single linguistic continuum, which was disrupted due to the expansion of Arvanitika (late 14^th^–late 16^th^) [50, 73–75]. It can be speculated that the term *φουρνός* could have also existed in Northern Euboea (Figs 6 and 7), but was replaced by the zoonym *brásca*, due to the arrival of Greek-speakers from mainland Central and Northern Greece, in an unspecified period after the 11^th^ century AD (Results 1: subchapter 4), and may have also affected the dialectal varieties of this part of the island [76] (Fig 9).

The toad zoonym(s) used by the archaic idiom of Old Athens and Attica has remained unknown. It is speculated here that the settlement of the Arvanites throughout Attica [50] and the linguistic shift of much of the original inhabitants led to the disappearance of the native names of *Bufo bufo* in the region, which were replaced by Arvanite ones (e.g. *thithëlopë*, *breshkë*, *bretkosë*) (Fig 2). Due to the proximity of Athens to Euboea and the Peloponnese, the zoonyms *φουρνός*-*φουρνία* or *βούζα* (*vúza*) (Results 1: subchapter 2.1.1) are likely to have been used in Old Athenian. Although speakers of the related and geographically adjacent Megaritic idiom [73, 74] participated in this study, all informants identified the toad as *βαρδακάς* (*vardakás* = frog), indicating that today’s Megarites cannot distinguish the common toad from the frog.

The presence of the term *φουρνόν* (*furnón*) in Pontus (Fig 5) (an Ionian colony in antiquity) is explained as a possible survival from the Hellenistic period, through successive phases of koineisation and the reinforcement of a Medieval Greek koine in the peninsula, prior to the 11^th^ century Turkoman settlements and the gradual linguistic Turkification of the region [51, 92–94]. Although the toad zoonyms used in other Greek-speaking linguistic islands of Asia Minor and beyond (dialects of Inepolis of Paphlagonia, Ano Amisos and Oenoe; also Crimaean, Cappadocian, Silliot and Pharasiot) are unknown, it can be assumed that the term *φουρνόν* survived in Pontus due to the geographical and cultural isolation of its inhabitants from the rest of the Greek-speaking population after the abovementioned Turkoman settlements, which contributed to the preservation of the Greek dialects of the region through the Empire of Trebizond and led to their "insularisation" [92, 94, 95]. The presence of the term *φουρνόν* in Romeyka, i.e. the dialectal varieties of Muslim Pontic speakers, which exhibit surviving archaic elements compared to the Pontic dialectal varieties of Christians [51], is of exceptional importance. This is because the zoonym *φουρνόν* may be a linguistic "fossil" from earlier times, possibly tracing back to an Ionian substratum, which may have passed into the local Hellenistic koine, and later into the Medieval Pontic common, the ancestor of modern Pontic. This finding is consistent with previous phylogenetic hypotheses regarding the evolution of Pontic Greek [51, 94]. The same applies to the descendants of the term *φρῦνος* in Andros and Euboea (*φουρνός*-*φουρνία*-*φουρνιά*), which were preserved in all subsequent phases where these dialectal varieties were absorbed by a Medieval Greek koine [96] (Fig 3). A decisive factor in the preservation of an ancient zoonym in these areas was their aforementioned isolation from the influence of mainland forms of Greek koine.

The zoonym *φουρνός* is also the source for the microtoponym *Φουρνιακό* (*Fourniacó* = where toads live) of the homonymous port in Zarakes, Euboea. The place name is justified, as the authors have observed that a large stream flows into the beach, which in spring attracts an impressive number of *Bufo bufo* even today. The microtoponym itself must be quite old, dating prior to the arrival of the Arvanites into the area in the 15^th^ century (the inhabitants of Zarakes traditionally speak Arvanitika). In fact, although the locals use the name *Fourniacó*, they do not know what it means, and instead employ the Arvanite zoonym *thithëlopë* to describe the toad (Results 1: subchapter 5.1.).

In summary, the terms descending from *φρῦνος* represent the oldest surviving zoonyms of *Bufo bufo* in the Greek-speaking world.

### 2.1. Βούζα (vúza) and likely derivatives

#### 2.1.1. Βούζα. Βούζα (vúza), βαλτόβουζα (valtóvuza), μπακόβουζα (bakóvuza), μπούζα (búza)

South-eastern Central Greece, northern and northeastern Peloponnese. **Vúzë** (in Arvanitika), northeastern Peloponnese.

For detailed distribution and additional derivatives, refer to S1 Text (chapter 2.1.2.).

*Discussion*. The term *βούζα* (*vúza*) stems from the erroneous beliefs that consider the toad to be a sucking parasite of the mammary glands of lambs, goats, and cattle (Results 2: subchapter 1). It is etymologically derived from the Hellenistic term *βυζίον* (*vuzíon* = tit, teat, breast), which evolved into the medieval *βυζί(ν) (vuzín*/*vizín*) [97, 98]. Folk beliefs that accuse reptiles or birds of being breast suckers [e.g. *βυζάστρα (vyzástra* = “titter”) for lizards in Cyprus, *γιδοβύζι* (*gidovýzi* = goat-tit) for nightjars, Caprimulgidae] can be found throughout the modern Hellenic world [32, 99], and evidently date to the Middle Ages at least, when Greek-speech was geographically unbroken (Fig 6).

The fact that *v****ú****za*, unlike the conceptually similar *βυζάστρα*, is not pronounced as *v****ý****za* indicates that the term resisted iotacism (i.e. the vowel shift where several vowels and diphthongs converged towards the pronunciation /i/) [100]. The phenomenon of iotacism began in Hellenistic times and is thought to have been completed in most of Greece by the 10^th^–11^th^ centuries AD [100]. However, several archaic idioms resisted this phenomenon, namely, those spoken in Old Athens, Aegina, Megara, Kymi. Boeotia, Locris, Mani and Tsakonia, which retained the pronunciation of -υ as /u/ instead of /i/ until recently [73, 100]. The distribution of these archaic idioms reflect the presence of a former linguistic continuum, which coincides with the areas that remained under Roman control during the Slavic migrations into the southern Balkans in the 6^th^–8^th^ centuries AD [48, 49, 78] (Fig 7), and are characterised by the smallest number of Slavic toponyms in mainland Greece (with the exception of Exo Mani) [14, 101]. A considerable amount of evidence suggests that this continuum was the result of a Constantinopolitan koine that formed around the 6^th^ century AD and became the dominant spoken language of the Eastern Roman Empire [96, 102–104]. The area covered by this linguistic continuum was later broken down and confined by the successive settlements of the Slavs, the Vlachs, and the Arvanites, but probably also by the creation of novel, simpler forms of spoken Greek, through language contact and assimilation of non-Greek-speaking populations (our hypothesis, described in more detail in Results 1: subchapter 3).

**Fig 10:**
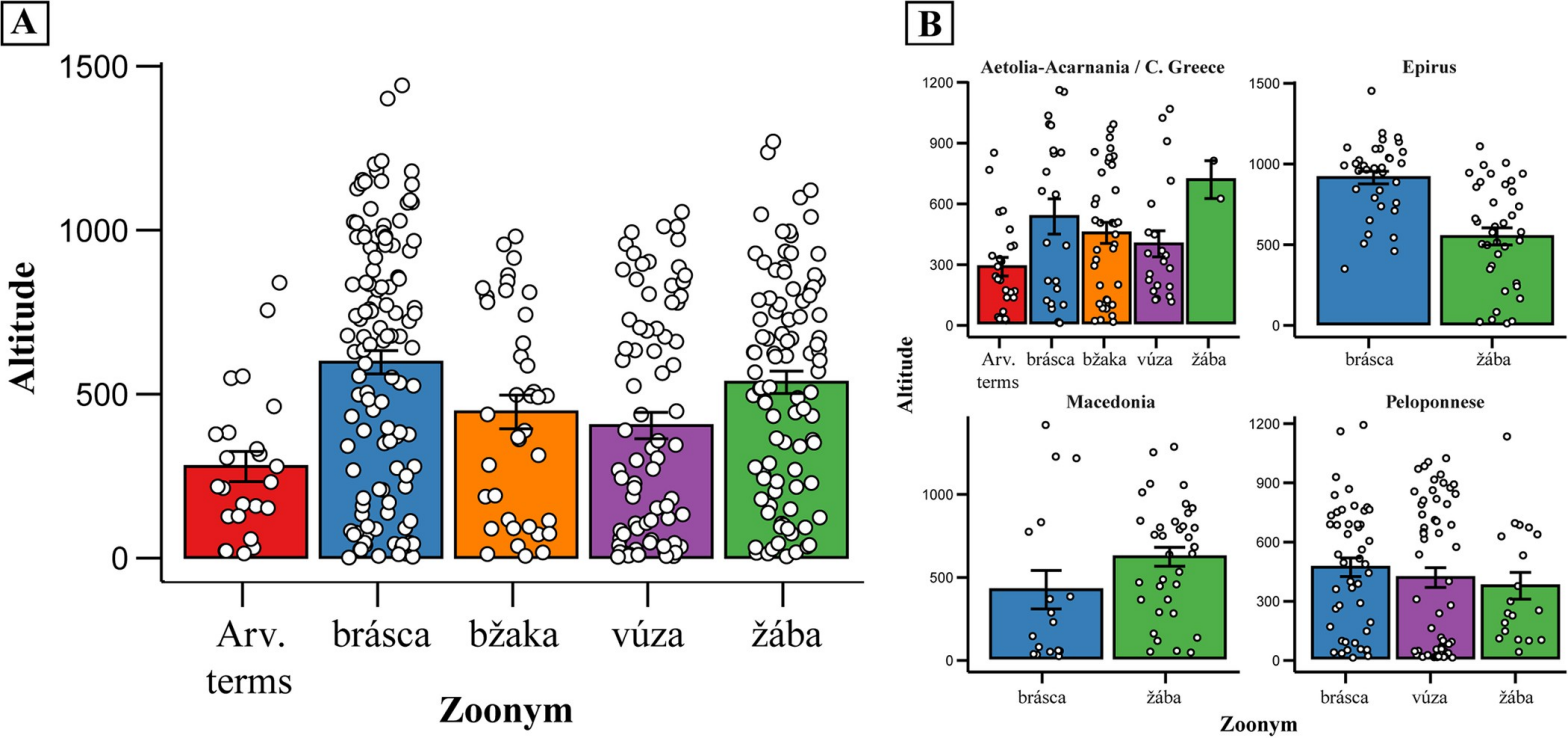
A) Altitude varies significantly relative to local zoonym usage in mainland Greece (Kruskal-Wallis test; χ2 = 22.886 at 4 degrees of freedom; p = 0.00013). Summary bars and error bars represent the mean ± standard error of the mean. B) Visualisation of the relationship between altitude and zoonym used in different parts of Greece. For both graphs, datapoints from Thessaly and Thrace were not used, as the terms *μπράσκα* (*brásca*) and *ζάμπα* (*žaba*), respectively, are entirely dominant in these regions. Datapoints from the Arvanite zoonyms of Epirus, Macedonia, and the Peloponnese, were also excluded, as they comprise a minor percentage of the total toad zoonyms used in those regions.

The distribution of the term *βούζα* in Boeotia, South Evrytania, Corinthia, and Achaia (Patras was an enclave of Greek-speech throughout the early Middle Ages [78, 101]) (Figs 2 and 7) also largely reflects the boundaries of this former linguistic continuum (Fig 7), as well as the areas where southern Greek varieties are spoken today or in the recent past [73]. Indeed, the disjunct range of this zoonym does not seem to be limited by geography or altitude, as it is found from mountains to sea level across a discontinuous landscape (Figs 2 and 10). This further affirms our suggestion that the factors that affected the range of *βούζα* were political (i.e. the Eastern Roman Empire) and not geographical, and emphasises the utility of toad zoonyms (and substrate terms in general) in identifying areas of linguistic and historical importance. Based on the information presented here, the most parsimonious explanation is that *βούζα* was the commonest toad zoonym in 6^th^-century AD southern Greece, before its distribution was broken up or completely replaced by Slavic and Vlach terms respectively (Figs 7 and 9). Whether *βούζα* represents a local development of early medieval Greece or an influence directly from the Constantinopolitan koine proposed by [96] is difficult to ascertain.

It is also interesting to note that the Arvanites of Boeotia and Peloponnese largely use the term *βούζα* (or the Albanised form *vúzë*, turned into a semivowel), undoubtedly as a loan from the Greek-language substrate, as has happened for many other terms [50].

The presence of the zoonym *βούζα* in Samos and Pylaia of Thessaloniki is certainly the result of colonisation and internal migration respectively, over the last centuries. Indeed, our informants from Pylaia, whose families have been native to their town for at least four generations, do not exclude the possibility that their grandparents heard the term *βούζα* from Peloponnesian immigrants who settled there in the early 20^th^ century. The cases of Samos and Thessaloniki provide further evidence for the importance of toad zoonyms as indicators of ethnological, linguistic, historical, and demographic phenomena.

#### 2.1.2. Μπζάκα (bžáka) and derivatives

**Μπζάκα (bžáka), Μπζιάκα (bziáka), Μπσιάκα (bsiáka), Μσιάκα (msiáka), Μπζάκλα (bžákla).** Western Greece (Aetolia, Acarnania, part of Evrytania, part of Phokida). **Μσάκα (mšáka), Μισάκας (misákas), Μουσάκα (musáka), Μπουσάκα (busáka), Μπουζάκα (buzáka)**. Ionian Islands: Cephalonia, Lefkada, Zakynthos. **Μπουσάκλα (busákla)**, **Βουσάκλα (vusákla)**. Lowlands of north-western Peloponnese (Ilia).

For detailed distribution and additional derivatives, refer to S1 Text (chapter 2.1.3.).

*Discussion*. The term *μπζάκα* (*bžáka*), which abides to northern Greek vocalism and phonetic rules [105–108], is perhaps the most unusual toad zoonym from a phonetic perspective. Younger speakers in the regions where it is found often struggle to achieve the palatalised /ž/, compared to older speakers, who pronounce it effortlessly. It is proposed here that the etymology of *μπζάκα* derives from the term *βούζα*, through the following development:

*βούζα (vúza* or *búza*) > *βουζιάκα* (*buz****iáka*) /** *βουζάκα* (*buz****áka*)** (augmentation through the suffix -**iáka/-aka**) > *μπζάκα* (*b****ž****áka*) (syncope of middle vowel /u/ and palatalisation of ζ to /ž/).

In modern Greek idioms, the palatalisation of consonants in words with the suffix– ια (ia) is a frequent phenomenon, especially the transformations -σ > -š and -ζ > -ž, e.g. καφάσια (kafásia) > καφάšα (kafáša), μαγαζιά (magaziá) > μαγαžά (magažá) [103, 107].

The forms *μσάκα*-*μψάκα*-*μσιάκα*-*μουσάκα-μπουζάκα* likely represent rebracketing of *μπζάκα* in the Ionian idioms, due to the difficulty in pronunciation that they cause to non-Northern Greek speakers when they first encounter these terms. Therefore, *μπζάκα* and its derivatives probably arrived in the Ionian Islands through Greek speakers from Aetolia-Acarnania. The zoonyms *μπουσάκλα* (*busákla*) and *βουσάκλα* (*vusákla*) (the suffix -akla functions as a re-augmentation) in north-western Peloponnese likely represent a secondary case of rebracketing of the Ionian variant *μπουζάκα*.

**Fig 11:**
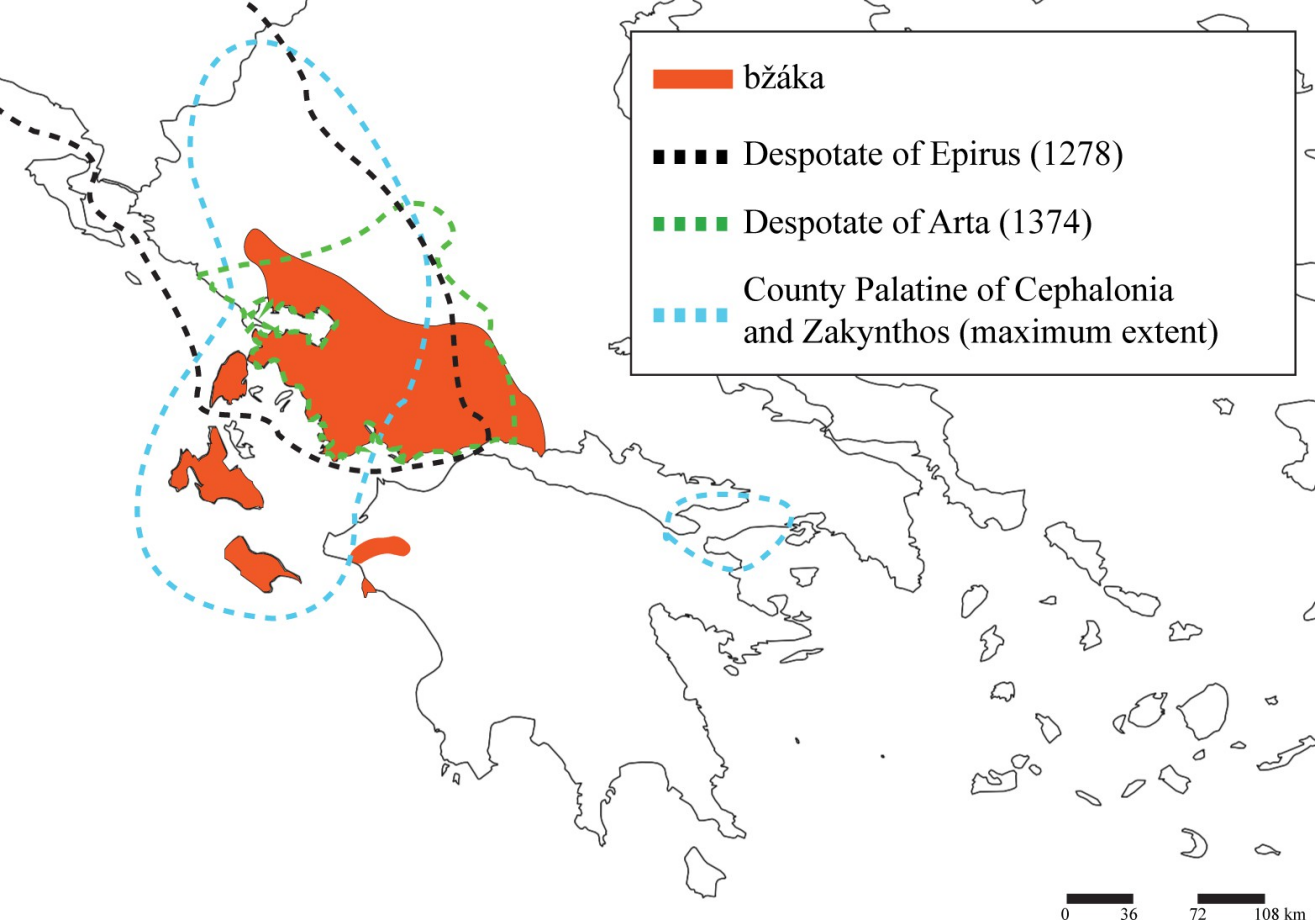
The distribution of the zoonym *μπζάκα* (*bžaka*) (orange) in relation to the boundaries of the Medieval states of Western Greece (black dotted line for the Despotate of Epirus, green for the Despotate of Arta, and blue for the County Palatine of Cephalonia and Zakynthos). The depiction of the states was based on [109–111].

The distribution of the zoonym *μπζάκα* and its derivatives is of particular historical interest. The term is endemic to south-western mainland Greece, with the following geographical boundaries: it includes the Ionian Islands with the exception of Corfu, a south-western zoonymic islet in lowland Ilia, a north-western extension to southern Epirus, a fairly continuous northern boundary along southern Pindos and western Central Greece from the Mornos River and westwards, while the coastal area of Vitrinitsa-Tolofon is its easternmost boundary (Fig 2). This distribution is unnatural, as the zoonym *μπζάκα* does not seem to be limited by the sea and geomorphology (e.g. altitude) (Fig 10B). It coincides, however, with the southern borders of the Despotate of Epirus, and the entirety of despotates that superseded it (County Palatine of Cephalonia and the Despotates of Arta and Angelokastro) at the beginning of the 13^th^ century AD. In particular, the distribution of *μπζάκα* and its derivatives accurately reflect the borders of the County Palatine of Cephalonia and Zakynthos (1185–1479), which had its seat in the Ionian Islands (except Corfu) in the 14^th^ and 15^th^ centuries, and under the Tocco family, the County Palatine subdued the neighbouring Despotates of Arta and Angelokastro, and temporarily acquired the wider area of Kyllini as well [109–111] (Fig 11).

Therefore, the zoonym *μπζάκα* most probably represents a local development of the Greek-speakers of Aetolia-Acarnania, and it was later transmitted within the borders of the Despotate of Epirus, the Despotates of Angelokastro-Arta, and the County Palatine of Cephalonia and Zakynthos. Relations between the County Palatine and neighbouring regions (e.g. the Duchy of Achaia) were often hostile [109, 111], and it can be assumed that movement outside its boundaries may have been restricted, and at least in one occasion, border-crossing provided the pretext for war (Battle of the Echinades) [109, 111]. These conditions played a crucial role in limiting the transmission of the term *μπζάκα*, which remains in the same area 500 years later (Figs 2 and 11). Accordingly, the distribution of the zoonym *βούζα* in Central Greece may have been restricted to its modern boundaries by the Grand Duchy (Signorie) of Athens (later under the Catalan State of Thebes [111]).

In summary, the following observations can be made regarding the term *μπζάκα*:

1. If *μπζάκα* evolved from the term *βούζα*, it testifies to the possible existence of the latter zoonym in medieval Aetolia-Acarnania, further strengthening the view that it was the dominant term in Greece from the 6^th^ century AD onwards.
2. The utility of toad zoonyms as evidence for the presence of historical networks and linguistic evolution receives further support.
3. The presence of the possible derivatives of *μπζάκα*, *μπουσάκλα* and *βουσάκλα* in Kyllini coincides with the temporary military successes of the House of Tocco in the 15^th^ century (Fig 8). If this etymology holds, it may indicate a small strand of migration from the County Palatine of Cephalonia and Zakynthos to the Peloponnese, if not merely linguistic contact or influence.

It is worth mentioning that the term *μπζάκα* is the source of the second microtoponym inspired by *Bufo bufo* that has been identified here. The river Ίναχος (Inachus, a tributary of the Acheloos in Aetolia) was formerly known to the locals as *Μπιζακός* (*Bizakós* = the one who contains many *μπζάκα*), due to the large number of toads that aggregated in its estuaries during the mating period.

A previous record of the term *μπζάκα* from an unspecified area of Corfu [32] is either incorrect, or represents a recent, localised introduction from Aetolia-Acarnania.

### 3. *Žaba* and its derivatives

#### 3.1. In Arvanites, Greek-speakers, and Vlachs. Ζάμπα (žába, zába, or zámba)

Disjunct distribution in Greek, Arvanitic, and Vlach-speaking areas; most of Epirus, Macedonia, Thrace, parts of western Peloponnese, pockets in Thessaly, Central Greece, and Central Peloponnese, all of Corfou. **Ζάμπια (zámbia or zápia).** Peloponnese (entire Mani peninsula). **Τζιάμπλακας (tziámblakas), τζάμπακας (tzámbakas)**. Pockets in Macedonia (area of Serres and Kilkis), very common in Thrace (Evros).

For detailed distribution and additional derivatives, refer to S1 Text (chapter 2.1.4.).

*3*.*1*.*2*. *In Slavophone areas of northern Greece*. **3.1.2.1. In Pomak. Ζάμπα (žába)**, **Ζέμπα (žéba)**. In all Pomak Muslim Slavophone areas of Western Thrace (Greek Thrace).

For detailed distribution and additional derivatives, refer to S1 Text (chapter 2.1.5.).

**3.1.2.2. In Slavophones (“ντόπια”, Slavomacedonian, Makedonski). Ζάμπα (žába)**, **Ζαμπαρόκ (žabarók).** Common in all Christian Slavophone areas of Greek Macedonia.

For detailed distribution and additional derivatives, refer to S1 Text (chapter 2.1.6.).

*Discussion*. Etymology from Slavic *žaba* (= frog) [112]. The entry of the term into the southern Balkans is readily dated to the 6^th^ century AD, during the first Slavic invasions and settlements into the region, which left much of the peninsula outside Roman administration, the Peloponnese specifically for 218 years, according to the *Chronicle of Monemvasia* (Fig 6) [77, 78]. It is likely that in early medieval times (600–1000 AD), *ζάμπα* and its derivatives were the dominant zoonyms of *Bufo bufo* in those areas of mainland Greece where *βούζα* was not found (Fig 7). The Slavic zoonyms of the toad may have been later restricted by the resurgence of the Roman Greek-speaking element, both due to local population growth and through resettlements by the Imperial administration in the 9^th^ century [13, 77, 78]. Subsequent settlements and expansions of other groups, such as the Vlachs, the Arvanites, and the Sarakatsani Greek-speakers, stimulated the introduction of yet more toad zoonyms (Results 1: subchapters 4–5).

Once again, toad zoonyms serve as excellent indicators of the linguistic, demographic, and historical processes of Greece, as the distribution of the derivatives of *ζάμπα* in the country reflects the areas which received intense Slavic settlement (e.g. large parts of Macedonia and Thrace), the areas which retain a significant Slavophone population to this day (e.g. northwestern Macedonia, northern Central Macedonia, parts of Eastern Macedonia, and Pomakochoria in Thrace) (Fig 8), but also the areas that have the highest number of Slavic toponyms nationwide (Epirus, Macedonia, Central-Western Greece, Western Peloponnese, Exo Mani) [14, 101]. Considering the above, the use of the term *žaba* and its derivatives is the result of long-standing linguistic contact between Greek-speakers and Slavic-speaking populations. The same pattern can be observed with other terms of ultimate Slavic origin that were incorporated into the agricultural vocabulary of mainland varieties of modern Greek (e.g. *λόγγος* = impenetrable forest, *βάλτος* = swamp, *σανός* = hay) [98].

The use of the term *ζάμπα* in a large part of lowland Ilia and the presence of “islets” of this zoonym in Arcadia and Messinia (Fig 2) indicate that there too the Slavic term was dominant, as they were places outside Imperial control [48, 49]. However, a linear persistence of this zoonym in Ilia directly from the first Slavic settlements cannot be confidently assumed, as the demographic continuity or discontinuity of the region further back in time is not historically attested. Furthermore, certain areas of the Western Peloponnese (lowland Achaia and Ilia and northern Messinia) experienced significant demographic changes in early modern times. Some of these include the movements of Arcadians and Arvanite nomadic pastoralists from Mt Panachaiko, who filled the population gap left by the exodus of Muslims after the Greek War of Independence [41]. It is possible that the zoonym *ζάμπα* was replaced in most of the Peloponnese in post-Medieval times, by the Vlach-derived term *μπράσκα* (*brásca*), which was introduced by Greek speakers from areas north of the Peloponnese (Results 1: subchapter 4). The same process may have taken place in Thessaly, where the Vlach and Greek-speaking population ultimately prevailed over the Slavophone element (Fig 2).

It should be stressed that the use of the term *ζάμπα* by the Greek speakers of south-western Greece is certainly not indicative of the survival of an active Slavic-speaking population in the region until recent times. More likely, in the uncontrolled part of the Empire during the Migration Period, the constant contact of Greek speakers with the Slavs, and the gradual assimilation of the latter into the Roman population [13, 14], may have led to the creation of a new, simpler Greek common speech (koine), which was characterised by numerous Slavic loans–one of which was *ζάμπα*. It should be emphasised that this hypothetical koine has left no written trace whatsoever, but effectively explains the high number of Slavic loans that characterises the south-western mainland Greek dialects, which are mainly related to rural life and occur in areas that remained outside Roman control for centuries. The existence of a distinct type of Greek koine in areas that bordered dense Slavic settlements satisfactorily explains why the archaic idioms of the Old Athens-Aegina-Megara-Euboea-Boeotia-Mani former linguistic continuum were confined only to areas which remained under Roman control during the Migration Period. It can be speculated that, in the western Peloponnese, this archaic linguistic continuum was disrupted by the emergence of a simpler koine, due to the radical change in the social and economic circumstances of the region and the extensive linguistic contact with the Slavs [13, 14, 77]. Indeed, past studies [e.g. 73, 100] have also suggested that the range of the Old Athens-Aegina-Megara-Euboea-Boeotia-Mani former linguistic continuum was disrupted or contracted by the expansion of a yet unidentified Greek variety that lacked the archaic characteristics found in the former.

Although this section focuses in Medieval Peloponnese (due to the larger number of historical sources), the linguistic situation described here may have applied to the entirety of western Greece, including Epirus. For example, the Slavic name of lake *Οζερός* (*Ozerós*) in Agrinio is particularly puzzling. According to geological data, the lake was formed after the 10^th^ century AD [113, 114], and one study [113] suggests that this toponym attests the persistence of Slavic speech in the area. Although several Slavic toponyms can be found in the environs of Agrinio [115], it is more likely that the name of the lake was given by Greek speakers who were linguistically influenced by earlier contacts with Slavic speakers. This is because the term *ozero* (= lake) is found only among Eastern Slavic languages (which were never spoken in Greece), while all South Slavic languages retain the /e/ of the proto-Slavic term *ezero [14, 112]. In fact, the toponym Οζερός has been etymologised as a modern Greek synaeresis of the singular type “*ο Εζερός*” (*o Ezerós*) > “*Οζερός*” (*Ozerós*) [14]. Therefore, it is suggested here that Greek-speakers may have used the loan *ézero* > *έζερος/εζερός* in their local idiom, which resulted in the synaeresis *ο Εζερός* > *Οζερός*. The situation in Agrinio adds further evidence to our hypothesis that in the wider area of southwestern Greece, a particular variety of Greek was spoken, which was influenced by contacts with Slavic-speaking populations. A Slavic-influenced Greek koine also explains why numerous Slavic terms (e.g. names of tools, adjectives, nicknames, zoonyms, phytonyms, personal names and surnames) survived and continued to be used in modern Greek idioms long after Slavic went silent in all parts of the mainland apart from Slavic-speaking parts of Macedonia and Thrace [13, 14, 77, 116]. Nevertheless, this novel hypothesis needs further investigation.

In several communities of Greece, toad zoonyms derived from *žaba* may represent more recent loans from various languages, although it should be stressed that the hypotheses presented here are highly speculative. These are the Maniots, the Arvanites of Epirus, and certain groups of Vlachs, who are discussed below.

Messinian or Exo Mani, a relatively small region of Greece, is characterized by the largest number of Slavic toponyms in the whole Peloponnese (over 400) [116]. In fact, a large percentage of the Slavic toponyms of Mani retain linguistic features which indicate Slavophone activity in the area until relatively recently [116]. Indeed, the region of Exo Mani (including Oitylo) and the southern slopes of the Taygetos range in general, served as refuges for the last Slavs of the Peloponnese, the Melingoi, who are mentioned as late as the 15^th^ century [14, 77, 101, 116–118] and probably survived as a distinct community shortly thereafter. The strong presence of Slavs in the area isolated the Maniots from the rest of the Greek-speaking world, and undoubtedly contributed to the preservation of their dialect, which features a distinctly archaic morphology, phonetics, and syntax [100].

All Maniots use the form *ζάμπια* (*zámbia* or *zápia*), which is endemic to the region and is a different interpretation of the zoonym *žaba* compared to the rest of Greece. This term is likely a loan from the Melingoi of the region, which is interesting, as the Maniots have preserved an archaic Greek dialect [100].

Genetics and historical records may provide some insights on how *ζάμπια* may have entered the Maniot vocabulary. The inhabitants of Mesa Mani have negligible Slavic ancestry [63], and this region is one of the candidate areas to have been first inhabited by the Maniots when they fled there during the Slavic invasions [119]. On the contrary, Exo Maniots derive a significantly higher proportion of their ancestry from the Slavs compared to Mesa Maniots [63]. Based on the above data, the most parsimonious explanation is that when the Maniots expanded from their probable cradle in Mesa Mani to the foothills of Taygetos (today’s Exo Mani), they encountered the last remaining populations of the Melingoi, with whom they interacted, and perhaps, eventually assimilated. Indeed, the Maniots probably maintained trade relations with the Melingoi (e.g. sale of their dairy products to the Romans [118]), and linguistic borrowings would not have been unknown. The possible contacts of the Maniots with the Melingoi may also account for the loan of the term *ζάμπια* to the Exo Maniots, who may have later transmitted this zoonym further south, to the inhabitants of Mesa Mani.

The possible use of other Slavic terms in the archaic Greek dialect of Maniot warrants further investigation, in order to estimate and date the extent of contacts with speakers of South-Slavic varieties, and possibly to geographically locate their interactions. It should be emphasised that one cannot ascertain whether the higher Slavic genetic contribution in the Exo Maniots is direct, i.e. the result of limited intermarriage with the Melingoi (before or after their hellenisation), or indirect, through later contacts with non-Maniot Greek-speakers, who also possess a non-negligible amount of Slavic ancestry [63, 64].

The use of *žaba-*derived zoonyms in Albanian is also particularly intriguing. Although most Albanians use toad zoonyms derived from proto-Albanian and vulgar Latin (Results 1: subchapter 5), certain south Albanian populations [36] and many Epirotan Arvanites instead use the Albanian form *zhabë*, or the Hellenised terms *ζάμπα*-*ζιάμπα*-*ζάππα*-*ζιάπα* (S1 Text, chapter 2.1.4.). Albanians had intense contacts with Slavs early on [120], yet the zoonym *zhabë* is unlikely to have entered the proto-Albanian lexicon as a loan, as it is only found in southern Albania–and proto-Albanian likely arose in the north of the country [120]. It is possible that *zhabë* entered southern Albanian idioms directly through Slavic assimilation or contact [121], as toponymy and genetics may suggest [37, 64, 122, 123]. However, this is highly unlikely, as there is no evidence of active Slavic speech in the area during the Albanian expansions [77, 124]. On the contrary, contemporary historical sources describe what is now southern Albania as an Eastern Roman stronghold [109, 124] that was characterised by a culturally and demographically dominant Greek-speaking population [125, 126]. The Greek dialect spoken in at least part of that region retains conservative characteristics most closely related to southern Greek idioms [38], which may be indicative of a long-standing presence of Greek in the region. Ergo, the likeliest explanation is that the term *zhabë* entered Albanian as a loan directly from the preceding Epirotan Greeks, who primarily use *žaba*-derived zoonyms to refer to the common toad even to this day (see below; S1 Text, chapter 2.1.4.). However, this hypothesis should be treated as tentative, as historical sources related to late Medieval Epirus are scarce [124].

Indeed, the use of zoonyms deriving from *žaba* predominates in Greek-speakers of lowland and medium altitude areas of Epirus (Figs 2 and 10B), serves as a testament to the past presence of dense Slavic settlements (sclaveniae) in the region, which is also mirrored by a large number of Slavic place names [14, 77, 101]. In contrast, in the highlands of Epirus, *žaba*-derived zoonyms are replaced by the Vlach term *μπράσκα* (Fig 10B), due to the historical settlement of Vlachs and later Sarakatsani, both of whom used that zoonym. However, *žaba* is not confined in the lowlands in the entirety of Greece, Macedonia being an exception (Fig 10B). This peculiarity is due to the Macedonian Vlachs, who inhabit high altitudes and have adopted the derivatives of *žaba* through their long-term interactions with present-day Slavic-speaking populations in the lowlands (Fig 2). In particular, the Meglenite Vlachs of Mount Paiko use the term *žabarók* (*žaba* + *rok*, Slavic augmentative suffix) (S1 Text, chapter 2.1.4.), while the Vlach term *μπράσκα* is reserved only for the tortoise (Results 2: subchapter 2).

Derivatives of the zoonym *žaba* have also been the source of at least two microtoponyms: the ravine *Ζάμπακας* (Zoni in Arcadia) and *Ζάμπλιακας* in Agios Konstantinos in Fthiotis [127].

### 3.2. Μπάτζιακας (bátziakas), μπάτσιακας (bátsiakas), μπατζαρόκα (batzaróka), μποζιάκας (boziákas)

Macedonia (primarily in Chalkidiki, pockets around Serres), pockets in Thrace and Thessaly (doubtful).

For detailed distribution and additional derivatives, refer to S1 Text (chapter 2.1.7.).

**Discussion** Zoonym that results from the metathesis of adjacent syllables in the *žaba*-derived term *τζάμπακας*, through the transformation ***τζ****ά****μπ****ακας* > ***μπ****ά****τζ****ιακας*. The zoonym *μπάτζιακας* is restricted to Macedonia (especially Darnakochoria and Chalkidiki) and Thrace (Fig 2). It is used by both Greek speakers and Vlachs. The disjunct distribution of this term, which is present in the environs of Thessaloniki and Evros (Trigono area), suggests that this term may have been more widespread, but was subsequently disrupted by Slavic or Ottoman settlements. This coincides with a former continuum of a Greek dialect that extended from the areas East of Thessaloniki all the way to Adrianople, and comprises the Greek spoken in southern Bulgaria (Eastern Rumelia), with the exception of the Black Sea Coast. A record of the term *μποζιάκα*ς from Euboea [32] is probably erroneous, or at least the result of recent migration, as it was not found anywhere on the island, despite the extensive sampling of the present study.

### 4. Μπράσκα (brásca) and its derivatives

#### Μπράσκα (brásca)

Dominant zoonym in Thessaly, mountains of Eastern Epirus, central-southern Peloponnese (excluding Mani peninsula), large parts of Argolis, central-northern Euboea, and Samos (Figs 1 and 2). Pockets across mainland Greece (Figs 1 and 2). **Μπρόσκου (bróscu), μπρουόσκα (bruósca), μπρουάσκα (bruásca)**, **μρουάσκα (mruásca).** Exclusively used by Vlach-speakers in Epirus and Macedonia. **Μπουράσκα (burásca).** Peloponnese (Argolis).

For detailed distribution and additional derivatives, refer to S1 Text (chapter 2.1.8.).

**Discussion.** The zoonym *μπράσκα* (*brásca*) and its derivatives stem from the Vlach term *broascã* (= frog), with a possible etymology from the term *broscus* of the late Latin *sermo vulgaris*, which may be related to the ancient Greek term *βροτάχος* (*vrotáchos*)*-βρότοκος* (*brótochos*) (= frog) [128, 129]. In fact, some Vlach villages of Thessaly, Epirus and Macedonia retain the probably archaic form *μπρόσκα (brósca)-μπρόσκου* (*bróscu*) (S1 Text, chapter 2.1.8.). The Hellenised form of *broascã*, *μπράσκα* (*brásca*)–which is the most widespread toad zoonym in Greece (Fig 2), results from synaeresis of the intermediate -o. The Vlach term *μπρουάτικου* (*bruáticu*) is likely a loan from Greek *βροτάχος* [129]. Although *μπρουάτικου* is used in Vlach for the frog, some of our informants also use it to name *Bufo bufo*, possibly due to misidentification.

The entry of the zoonym *μπράσκα* into Greece is easily traced to the beginning of the 9^th^ century AD, perhaps even a little earlier, with the first mass settlements of Vlachs. The Vlachs (or Armân, Rămăn) are a Romanised Balkan people (probably linguistically related to the proto-Albanians [5–12]), who speak one of the four Eastern Balkan Romance languages and were first mentioned in Greece in AD 976, in an area between Kastoria and Prespa [130]. A later source is the *Strategikon of Cecaumenus*, which mentions that in 1066, the Vlachs of the Thessalian plain rebelled against the Emperor Constantine I Doukas, due to the high taxes he imposed upon them [131].

The Vlachs are described as nomads of the mountains of Thessaly [132] and “Bulgaria” [= Pindos range and the mountains of Western Macedonia (Florina, Kastoria, Bitola)], who often plundered the local Romans [88], but some of them served as soldiers of the Empire against its enemies [132]. The existence of settled Vlach villages is also attested in the same period [132]. The fact that as early as 1160 Thessaly was also called Vlachia [88], and a little later as Great Vlachia (or Great Wallachia) [89], suggests that their demographic and cultural contribution to the region was very significant.

The Vlachs’ transhumant way of life, combined with the fact that they constituted a significant (and in some places dominant) demographic element in a large area of the southern Balkans, brought them for centuries in direct contact with all other populations of mainland Greece (excluding the Peloponnese) [42]. From the very beginning of their presence in Greece, and for more than 1000 years, the Vlachs coexisted and had trade relations with the settled Romans, Arvanites, and Slavs, exchanging ideas, beliefs [91], and–as is it is shown here, words. In fact, the exonym “Vlachs” became inextricably linked to nomadic pastoralism, with the result that the settled agricultural populations came to refer to all transhumant shepherds as Vlachs, regardless of their ethnic origin [133]. For example, the Maniots, probably due to the absence of nomadic pastoralism in their homeland, used the term *βλάχος* (= vlach) pejoratively to describe all Peloponnesian pastoralists and even the native Corsican herders in the Maniot colony in Cargèse, Corsica, which was established in the 17^th^ century [134, 135].

Taking the above data into account, it is not surprising that the distribution of the term *μπράσκα* in central and northern Greece largely reflects the boundaries of the Great Vlachia of the 11^th^ and 12^th^ centuries (Fig 9). The data presented here also enables the identification of the wider geographical area where intense contact between Vlach and the local varieties of spoken Greek took place. As a result, the local Greek-speakers have adopted the term *μπράσκα* as the exclusive zoonym for the common toad, which is not the case in Vlach, where this zoonym retains multiple meanings (it refers to either the frog, the toad, or the tortoise; Results 2: subchapter 2, Table 1).

**Table 1:** 

Zoonym	Language	Meaning	Region
**The tortoise as a saddled toad or frog:**			
bróscu cu smáru	Vlach	saddled frog (= tortoise)	Metsovo
bróscu	Vlach	frog	Metsovo
**The toad as a “naked” tortoise:**			
μπράσκα (brásca)	Greek	tortoise	Leptokarya (Zagori)
μπράσκα ξεσαμάρωτη (brásca ksesamároti)	Greek	tortoise without a saddle (= toad)	Leptokarya (Zagori)
ζαρκοχελώνα (zarkochelóna)	Greek	naked tortoise (= toad) [from *ζάρκ-* (*zark-* = naked in the Epirotan, Thessalian, and Ionian idiom, of unknown etymology) + *χελώνα* (*chelóna* = tortoise)]	Leptokarya (Zagori)
μπάκακας ξεμπλέτσωτος (bákakas kseblétsotos)	Greek	naked frog (= toad) [from the local idiomatic term *ξεμπλέτσωτος* (*ksemblétsotos* = naked)]	Negades (Zagori)
**Other differences in meaning:**			
bróscu	Vlach	Male tortoise (= toad)	Some Vlachs in Macedonia (Kilkis) and Epirus (Aetomilitsa, Metsovo)
brósca	Vlach	tortoise	Some Vlachs in Macedonia (Kilkis) and Epirus (Aetomilitsa, Metsovo)
brásca	Meglenite Vlach	tortoise	Paiko
žabarók	Meglenite Vlach	toad	Paiko
bruáticu	Vlach	frog or toad	Macedonia (Dion)
brásca/bruásca	Vlach	tortoise	Macedonia (Dion, Serres)
φαρφαλιώπα and derivatives	Arvanitovlach	toad	Arvanitovlach settlements
bruósca/bróscu/bruásca/brusóca	Arvanitovlach	tortoise	Arvanitovlach settlements
bruáticu/brútica	Arvanitovlach	frog	Arvanitovlach settlements

**Fig 12:**
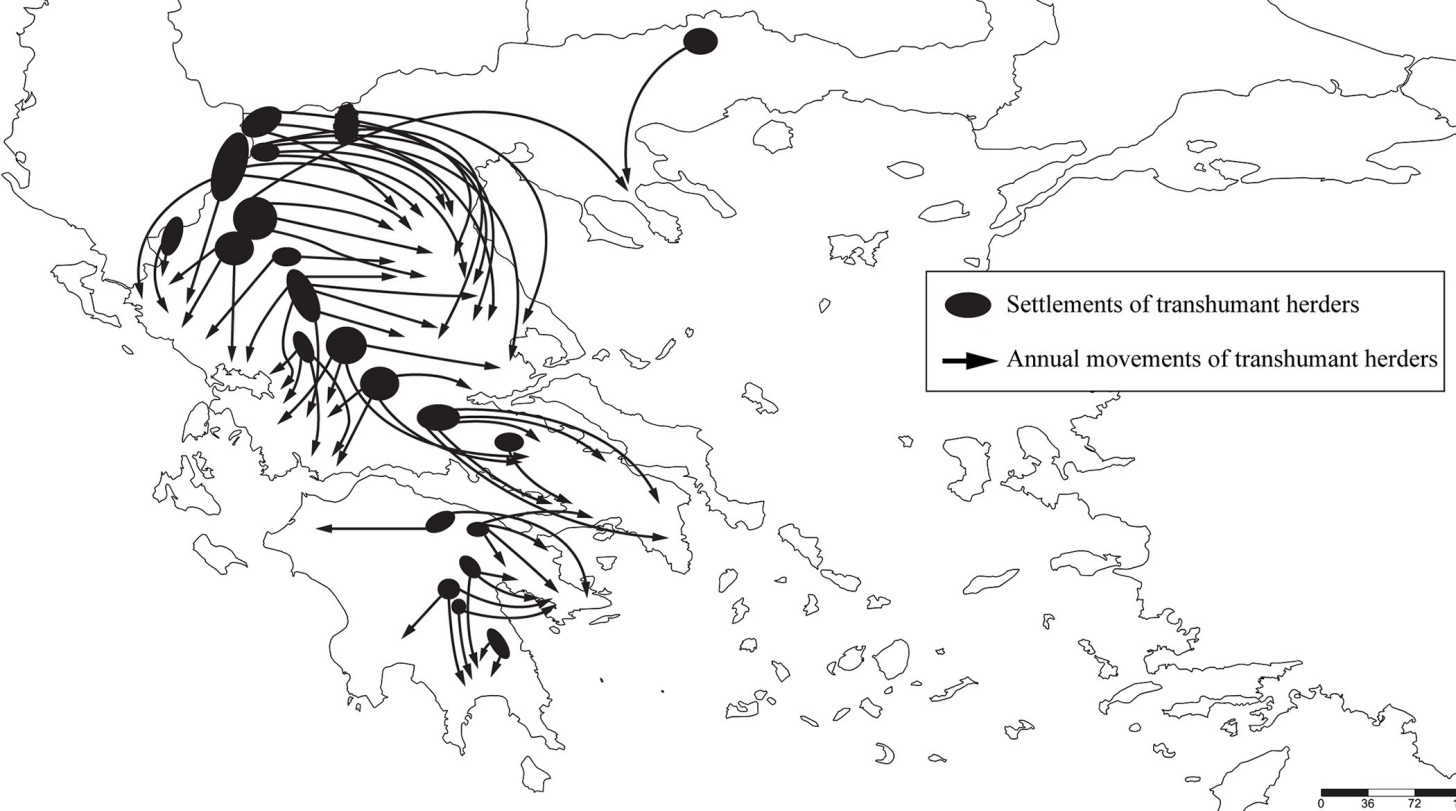
Representation of the annual movements of the transhumant herders of Greece (regardless of ethnicity or language) in 1981. The map was based on [90]. Movements of transitional breeders from Eastern and Central Macedonia and Thrace are not depicted, as their annual journeys greatly diminished or disappeared in those areas following the creation of the northern border of Greece in 1913–1920, the settlement of the exchanged Greeks of Asia Minor and Thrace (1923–1926), and the emigration of many Vlachs to Romania (1926 onwards) [42, 91].

The distribution of *μπράσκα* is also correlated with the settlements of Vlach populations outside Thessaly according to ethnological maps of the 19^th^ and 20^th^ centuries [79]. In particular, the linguistic islands of the term *μπράσκα* in Messolonghi, Xiromero, Karpenisi, and Preveza (Fig 2) testify to the settlement of Vlachs, Sarakatsani Greek-speakers (see below), and pastoralists from Tzoumerka in those places, which served as winter pastures for their herds (Fig 12). Indeed, groups of Vlachs settled permanently in lowland areas of Greece multiple times independently [42] and contributed to the further spread of the term *μπράσκα* to Greek speakers and Arvanites. Based on the above, the term *μπράσκα* can be used to map Vlach settlements, but also areas inhabited by Greek and Albanian speakers with lexical borrowings from Vlach.

The Sarakatsani, who are Greek-speaking nomadic pastoralists attested since the 17^th^ century [136, 137], serve as the main example of the transmission of the term *μπράσκα* by non-Vlachs. With distant origins in Agrafa and southern Pindos in general (i.e. within the Vlachs’ sphere of influence) [133, 136, 137], the Sarakatsani were the most prominent nomadic pastoralists in the Balkans alongside the Vlachs. Their movements covered a wide area that covered most of central and northern Greece, including Argolida, Attica, Euboea, as well as areas outside Greece (Albania, North Macedonia, Bulgaria, and parts of Eastern Thrace and northwestern Asia Minor) [133, 136, 138, 139]. The present research shows that *μπράσκα* is the dominant (and perhaps the only) term used by Sarakatsani throughout Greece to describe the common toad, and it is hypothesised that they contributed significantly to the dispersal of the term in places where Vlachs never ventured. This hypothesis is confirmed by the fact that while the Slavic term *žaba* is dominant in Thrace, “islets” of the zoonym *μπράσκα* can be found in the Sarakatsani village of Loutra in Evros, as well as in other villages of Eastern Macedonia and Thrace where Vlachs are absent (S1 Text, chapter 2.1.8.). The Sarakatsani are probably also responsible for the transmission of the term in the Peloponnese–at least in part, as will be shown below.

The presence of the term *μπράσκα* in a large part of the Peloponnese is intriguing, as there is no evidence of Vlach settlements in this area [133], although small-scale migrations cannot be excluded. The indirect dispersal of the term by Greek-speakers coming from the north is the likeliest explanation, as northern Peloponnese (Chelmos, Zireia, lowland Argolida and Corinthia) was settled by some of the Sarakatsani, and received additional Greek-speaking herders from central Greece [140]. The Peloponnesian farmers called those herders as *Σκηνίτες* (*skinites* = tent-dwellers), *Ανεβοκατεβάτες* (*anevokatevates* = up-and-downers), *Ρουμελιώτες* (Roumeliots), Vlachs (i.e. nomadic herders), or, in writing, *Βλαχοποιμένες* (*Vlahopimenes* = Vlach-herdsmen), due to their transhumant way of life [133, 140, 141]. Although the origins of these herdsmen have been widely debated, analysis of historical sources, their surnames, and their oral traditions suggests that at least some of them hail from the Sarakatsani [133, 142].

During spring and summer, many Arcadian herdsmen would use Messinia as a winter refuge for their flocks [140, 141]. Frequently, entire villages of “*Σκηνίτες”* and other groups of mountain herdsmen migrated to neighbouring Messinia, as well as to Argolida and Laconia [140]. The distribution of the term *μπράσκα* accurately reflects the annual routes of transhumant herdsmen of the Peloponnese [90] (Fig 12), which took place in areas that are known to have received Arcadian settlers [140]. Moreover, many formerly Arvanite-speaking populations of the Peloponnese (e.g. in Argolida and Laconia) also use the term *μπράσκα* or *μπουράσκα*, apparently as a loan from Greek-speaking farmers and/or from Sarakatsani and Arcadian transhumant herders, who overwintered there. The term *μπράσκα* has also been adopted by Tsakonian, one of the most aberrant Helladic languages [97, 100, 143], which is spoken in about 10 villages of eastern maritime Arkadia (e.g. Kastanitsa, Prastos, Leonidio, Tyros), but had a much wider distribution in the recent past [97]. This adoption is almost certainly a loan from neighbouring speakers of modern Greek varieties.

Based on the above, the *Σκηνίτες* of Arcadia and the Sarakatsani can be considered as the *de facto* transmitters of the term in the Peloponnese. Although the first *Σκηνίτες* are attested in the Peloponnese as early as 1690 [144], the influx of Greek speakers from the north, who probably used the term *μπράσκα*, may have started earlier. This hypothesis is difficult to verify, as these periods are characterised by large historiographical gaps.

The inhabitants of the village of Lekka in Samos, which was settled in 1581 by Peloponnesian Greeks and Arvanites [145], also use the term *μπράσκα* [32]. If the first settlers of Lekka used this zoonym, then it could provide us with a possible *terminus ante quem* for the first settlements of populations from northern Greece into the Peloponnese. However, it should be stressed that this scenario is hypothetical, as the term *μπράσκα* could have been introduced to the inhabitants of Lekka through interactions with settlers from other parts of Greece. Identifying the linguistic influences in Samos is complex, as the island had remained uninhabited until the 16^th^ century, when it started being settled from mainland Greece, the surrounding islands and Asia Minor [145]. This study has identified several villages of Samos which use mainland toad zoonyms, such as *μπράσκα* (Pagonda, Paleokastro, Chora) and *βούζα* (Pagonda) (Fig 2), which are indicative of linguistic influences from the Peloponnese, central and northern Greece.

The Karagounides, the main group of Greek-speaking farmers of lowland western Thessaly, should also be mentioned. The origin of the Karagounides–who were first mentioned in the early 17^th^ century–is enigmatic [146]. A plethora of theories suggest origins either from native Greek speakers, from Hellenised Arvanites or from Arvanitovlachs. However, neighbouring Vlach populations refer to the Karagounides as "*Greči*" (= Greeks), suggesting that this has always been their ethnic identity [146]. As for the name they use for the common toad, the predominant zoonym among the elderly is the Karagoun-specific *μπράχτσα* (*bráhtsa*) (or *μπράτσα* in Vlochos) (S1 Text, chapter 2.1.8.). Younger Karagounides use the term *μπράσκα*, probably as a result of contacts with neighbouring Greek-speaking groups. The Karagounides of the villages of Marathea, Palamas, and Proastio also use the Slavic term *ζάμπα* as well as *μπράσκα*, which is probably evidence of an earlier linguistic stratum of local Greek that used this term.

The presence of the term *μπράσκα* throughout Central and Northern Euboea merits further discussion. As there is no evidence of large-scale Vlach settlements in Northern Euboea, it is hypothesised that the zoonym *μπράσκα* reached the island through mainland Greek-speaking mediation, as it likely did in the Peloponnese. Although Euboea is more accessible from the mainland via Chalkida, it is assumed that the Greek-speaking settlers in question did not use this route. In Boeotia, which is the mainland coast opposite Chalkida, the dominant toad zoonym is *βούζα*, a term that seems to be absent from Euboea (Fig 2). Therefore, the most parsimonious explanation requires the migration of Greek-speaking settlers directly from Thessaly or north-central Greece to North Euboea, introducing the term *μπράσκα* (Fig 9, white arrow). The probable absence of tsitacism (affricating of the palatal stop /k/ to /tʃ/ or /ts/ before the short vowels /i/ or /e/) in northern Euboea already in the 15^th^ century–in contrast to the rest of the island at that time (including Chalkida) [76]–argues for a different linguistic (perhaps even demographic) influence in this part of the island.

### 5. Arvanite zoonyms for the common toad

#### 5.1. Θιθιλιώπα (thithiliópa), Σισελιώπα (siseliópa), Τιθλιώπα (tithliópa), Μπιθλιώπα (bithliópa), Χλιώπα (hliópa), Θυθλιώπα (thithliópa), Θλιώπα (thliópa)

Used primarily by Arvanites in Epirus (especially towards the coast) and pockets in Euboea. Occasional use by Greek-speakers in pockets in Epirus.

For detailed distribution, refer to S1 Text (chapter 2.1.9.).

*Discussion*. Etymology from Albanian *thithëlopë* = cow sucker [120]. This zoonym entered Greece with the first Arvanite settlements in the 14^th^ and 15^th^ centuries AD. Although most users of this term are of Arvanite origin, the Greek-speakers of western Tzoumerka may have acquired the term from the neighbouring Cham Albanophones, or *thithëlopë* may represent a substrate term in a recently Hellenised population (especially in the southern parts of the region of Ioannina, Preveza, and Thesprotia [41]).

#### 5.2. Μπρέσκλα (bréskla)

Primarily used by Arvanites in Epirus and Central Greece (mainland and Euboea).

For detailed distribution and additional derivatives, refer to S1 Text (chapter 2.2.1.).

*Discussion*. Etymology from Albanian *breshkë* = tortoise, which was loaned into proto-Albanian from the Latin term *broscus*, meaning toad [120]. This zoonym entered Greece with the first Arvanite settlements and is still used primarily by the speakers of this language, and occasionally by their Greek-speaking neighbours as well.

#### 5.3. Μπερτικοσα (bertikósa), μπρτόσκα (brtóska)

Exclusive use by Arvanites in Central Greece. Pockets in the Cyclades (Andros), Peloponnese (Argolis) and Thrace (Evros).

For detailed distribution, refer to S1 Text (chapter 2.2.2.).

*Discussion*. Etymology from the Albanian *bretkosë* = frog, ultimately from Greek *βροτάχος*-*βρόθακος* [120]. All users of this term are Arvanites. Some of the Arvanites of Evros use the term *μπρτόσκα* (*brtóska*), or its entirely syncopated term *μπρτσκ* (*brtsk*), which in the rest of Greece was found only in Askri of Boeotia, where it denotes the tortoise (S1 Text, chapter 2.2.2.). It should be noted that, among recently monolingual Greek-speaking descendants of Arvanites, the term *μπερτικόσα* has become the near exclusive zoonym for the toad (even though it originally referred to the frog). In the same way, the originally Vlach term *μπράσκα* has become the principal toad zoonym among the Greek-speakers of Thessaly and parts of Central Greece and the Peloponnese, even though it retains multiple meanings among native Vlach speakers (Results 1: subchapter 4; Results 2: subchapter 2, Table 1).

#### 5.4. Μπακούα (bakúa)

*Central Greece*: Markopoulo in Mesogaia (Arvanites).

*Discussion*. Unknown etymology, possibly from Greek *μπάκακας* (= frog).

#### 5.5. Μπαρακότσκα (barakótska)

*Macedonia*: Drosopigi and Flambouro in Florina (Arvanites).

*Discussion*. Unknown etymology. The suffix -οτσκα is indicative of Slavic influence.

#### 5.6. Μτρμούμα (mtrmúma)

*Cyclades*: Andros (former municipality of Gavrio, i.e. Ammolochos, Varidi, Vitali, Gides, Kalyvari).

*Discussion*. A zoonym endemic to some Arvanite villages of Andros, in the former municipality of Gavrio. *Μτρμούμα* (*mtrmúma*) is an Arvanite kinship term, which means maternal aunt. In this case the zoonym reflects the similarity between the characteristic behaviour of the toad when it encounters a house wall (it usually attempts to climb it, placing its front legs over its head) and the hand motions of the *μτρμούμα* when hanging clothes to dry.

### 6. Toad zoonyms of more restricted distribution

#### 6.1.1. Greek zoonyms referring to the defensive inflation of *Bufo bufo*

Apart from the parotid glands, which secrete toxins, toads have only one other defence mechanism. When threatened, toads lift their legs and inflate their body to appear as more formidable opponents against would-be predators [147]. This behaviour is the basis of at least five Greek zoonyms.

#### 6.1.2. Ασκουβάζα (askuváza*)*

*Peloponnese*: Eastern Corinthia (Chiliomodi, Agionori, Klenia, Piada, Stefani), Argolida. **Skuvázë / σκουβάζα (skuváza)** (both forms exclusively used by Arvanites). *Peloponnese*: Angelokastro, Arachnaio (Cheli), Kalloni Trizinias.

*Discussion*. Possible etymology from the term *ασκός* (ascós = leather bag used to store liquids; alternatively, the bagpipe). It was not possible to offer a reliable etymology for the second component of this zoonym (-vaza), although it could potentially derive from *βούζα*, even though the authors are unaware of the assimilation of - ου to -α in Modern Greek. Based on the first component of this zoonym, it likely means bag-toad or bagpipe toad. This particular zoonym is endemic to Argolida and Eastern Corinthia, and is used both by Greek-speakers and Arvanites (among the latter with the form *skuvázë*, Hellenised as *σκουβάζα*).

The zoonym *ασκουβάζα* probably represents a local development of the Greek-speaking linguistic islet of Argolida and Corinthia, which was surrounded by an Arvanite "sea" [41, 50] (Fig 8B). If our hypothesis is valid, then *ασκουβάζα* may date much earlier than the 15^th^ century, before the first Arvanites settled the area. The term *ασκουβάζα* is mentioned as part of the vocabulary of the Arvanites of the Greek fleet (mainly from Hydra, Poros, and Spetses) by [148].

The Arvanites of Methana seem to have resisted the influence of the Greek term *ασκουβάζα* and have instead retained Arvanitic term *μπερτικόσα* (S1 Text, chapter 2.2.2.; Fig 2), while some villages of central and western Argolida use the zoonyms *μπράσκα* and *μπουράσκα*, probably influenced by the Arcadians and Sarakatsani who overwintered there (SI Text, chapter 2.1.8.; Fig 12).

#### 6.1.3. Μπασκαφούτα (bascafúta)

Peloponnese, endemic to the region of Trifillia in Messinia. **Ασκουφούτα (ascufúta)**: Peloponnese, single location in Arkadia (Stolos). **Μπρασκαφούτα (brascafúta).** Peloponnese (Messinia), doubtful isolated cases in Epirus and Central Greece (Euboea).

For detailed distribution, refer to S1 Text (chapter 2.2.3.).

*Discussion*. Disjunct distribution, covering a large area in Messinia and a small pocket in Arkadia (Fig 2). Possibly a combination of *μπράσκα* and a zoonym related to *ασκουβάζα* (perhaps *ασκουφουτα*-*ασκαφούτα*), that refers to the defensive inflation of toads. If this hypothesis is accurate, then zoonyms related to *ασκουβάζα* and *ασκουφουτα*-*ασκαφούτα* may have had a much broader distribution in the Peloponnese in the past.

#### 6.1.4. Σακούδα (sakúda)

*Peloponnese*: villages of the plain of Ilia (between Kato Achagia and Gastouni, e.g. Agnanta, Smila, Pelopio), as well as in the adjacent highlands (Divri) and the plateau of Kapeli (Koumanis).

*Discussion*. Τhe zoonym *σακούδα* (*sakúda*) originates from the ancient Greek term *σάκκος* (sákkos = sac), which according to Beekes [65] is a Phoenician loan. This zoonym is endemic to the plain of Ilia and the adjacent mountains.

#### 6.1.5. Σακκούτα (sakkúta). Calabria (all Griko villages)

*Discussion*. The zoonym *σακκούτα* (*sakkúta*) is conceptually and etymologically the same as *σακούδα*. Our informants claim that this is the primary toad zoonym used by the Griko speakers of Calabria, instead of the Italo-Romance terms *buffa* and *rospo*.

The population of Southern Italy has strong genetic connections to those of the Eastern Mediterranean, due to Greek colonisation in archaic times [149], as well as to later demographic events during Imperial Roman and Medieval times, which transformed the entire peninsula [150, 151]. The Greek-speakers of Apulia and Calabria speak two Greek dialects that date back to the early Middle Ages [152, 153], but retain numerous influences from a native Doric substrate [153–155]. The origin of the term *sakkúta* cannot postdate the 12^th^ century AD, which is when the Eastern Roman Empire lost Southern Italy to the Normans, and influences from non-Italiot Greek disappeared.

If *σακκούτα* was an influence from the Eastern Roman Empire, it obviously must have developed before the 12^th^ century AD. The Italiot *σακκούτα* and the Peloponnesian *σακκούδα* may have evolved independently, although one cannot exclude the possibility of a common origin for the two terms. Whether *σακκούτα* or other toad zoonyms are used in Apulia remains to be found.

#### 6.1.6. Φουσάκλα (fusákla)

*Peloponnese*: valley of Nezera, Lakkomata Erymanthou, Western Achaia, Chrysopigi-Spodiana of Achaia.

*Discussion*. The term *φουσάκλα* (*fusákla*) derives from the ancient Greek *φῦσα* (*phúsa* = bladder, pouch, bubble, wind) and the augmentative suffix -ακλα. It is probably genetically related to the ancient Greek toad zoonym *φῡσᾰλος* (*phúsalos =* the inflated one) [156], which is also attested as a personal name in an inscription found in Naupactus in the 2^nd^ century BC [157]. Indeed, the preservation of the /u/ pronunciation, instead of the iotacised /i/ pronunciation, suggests that this zoonym is particularly ancient, just as is the case with *βούζα*. The toad is also named *φύσαλος* in Darvaris’s Encyclopedia [158], but this is probably a deliberate archaism, rather than a term in everyday use.

The strong localisation of *φουσάκλα* to the region of Nezera and its environs, an enclave of particular linguistic and anthropological interest, deserves further investigation.

This is because the villages of Nezera owe their name to the Slavic tribe of Ezerites, who are reported to have settled the region in the 9^th^ century after a failed rebellion, which led Leo Scleros to expel them from their homeland in Mt Parnon and the lowlands of Evrotas [77]. The presence of the zoonym *φουσάκλα* –a Greek term that potentially dates to Late Antiquity–in the historically Slavic-settled Nezera might be surprising. However, the linguistic and demographic evolution of the Nezera and their environs over the past 1000 years is obscure, as they are not mentioned in any historical sources. As anywhere else, one cannot assume traces of a Slavic linguistic substrate in the area in the absence of compelling evidence.

Some of our Achaian informants outside Nezera (e.g. in Aigio) were familiar with the term *φουσάκλα* or *φουσάκα* but could not remember where they heard it. This suggests that this zoonym may be more widespread than what is currently shown in Fig 2. It is possible that limited nomadic pastoralism from Nezera to the lowlands (or vice versa) has contributed to the dissemination of *φουσάκλα*.

The only other toad zoonym encountered in Nezera is *βούζα*, likely a loan from neighbouring populations.

### 6.2. Terms related to the sucking of the mammary glands of livestock (cattle, goats, sheep)

#### 6.2.1. Among Greek speakers

*6*.*2*.*1*.*1*. *Βούζα (Results 1*: *subchapter 2*.*1*.*1*.)

*6*.*2*.*1*.*2*. *Μασταράς (mastarás)*. *Epirus*: Konitsa. *Macedonia*: Kalirachi in Grevena.

**Discussion**. Etymologised from the term *μαστός* (*mastós* = breast, teat) + αράς (augmentative suffix). In the same areas it is also known as *γιδοβύζι* (*gidovýzi* = goat tit, a zoonym otherwise given to the nightjar, a bird of the Caprimulgidae family).

6.2.1.3. Αγελαδοβύζα (ageladovýza) / Βυζογελάδα (vyzogeláda). *Epirus*: Petra in Zagori.

**Discussion.** From *αγελάδα* (*ageláda* = cow) + *βυζί* (*vyzí* = tit), as a semantic calque from Albanian *thithëlopë* (Results 1: subchapter 5.1.).

#### 6.2.2. In terms of potential Vlach origin

*6*.*2*.*2*.*1*. *Μπρουζογελάδα (bruzogeláda) / μπρουζουγέλα (bruzugéla)*. *Epirus*: Tristeno. **Μπρουζογέλαδο (bruzogélado).** *Epirus*: Itea in Zagori.

**Discussion.** Etymologised from the local term *μπρόζγου*/*μπρούσγου* (*brózgu*/*brúzgu* = frog, from Vlach *bróscu*) + *αγελάδα* (= Greek for cow), as locals believe that the toad sucks the milk of cows at night. The term *μπράσκα* is reserved only for the tortoise in these villages.

#### 6.2.3. In Arvanites

*6*.*2*.*3*.*1*. *Θιθιλιώπα and derivatives (Results 1*: *subchapter 5*.*1*.*)*.

### 6.3. Terms related to the size and obesity of the toad (among Greek speakers)

**6.2.3.1. Βατραχομάνα (vatrachomána).** *Central Greece*: Phocis.

*Discussion*. From the terms *βάτραχος* (*vátrachos* = frog) + *μάνα* (*mána* = mother, as an augmentative metaphor). Conceptually similar to *μπατσιακομάνα* (S1 Text, chapter 2.1.7.) and *κουβακομάνα* (S1 Text, chapter 2.2.4.). The component -μάνα is used as an augmentative suffix for other zoonyms as well, e.g. *καβουρομάνα* (*kavuromána* = mother-of-crab, referring to unusually large edible crabs).

**6.2.3.2. Χοντράδι (hondrádi).** *Central Greece*: Attica (Thoriko, Lavrio).

*Discussion*. From the adjective *χοντρός* (*hondrós* = fat), due to the obese appearance of the common toad, especially females during the reproductive period.

### 6.3. Greek zoonyms of unknown or uncertain etymology

#### 6.3.1. Τσιόφλακας (tsióflakas)

*Turkey*: Livissi = Kayaköy. **Τσόφλακας (tsóflakas)**. *Turkey*: Makri = Fethiye.

*Discussion*. The common toad has a prominent role in the traditions of the Greek-speakers of Livissi (now Kayaköy) and Makri (now Fethiye) in south-western Asia Minor, who speak an archaic dialect related to Cypriot and Dodecanesian Greek [92]. The term *τσιόφλακας* is mentioned in numerous children’s tales and beliefs (Embirikos, L., personal observations, [159]). Its cultural role is so strong, to the extent that the descendants of the refugees of Livissi and Makri in Kato Tithorea (Kifissochori) and Euboea (Farakla, Kirinthos) still retain the zoonym *τσιόφλακας*, even though 2–3 generations have passed since their forebears fled from their ancestral homes.

Although the etymology of this particular zoonym is unknown, it may be derived from the Medieval term *τσόφλι* (*tsófli* = shell, husk) [98], due to the dryness of the toad’s skin compared to that of the frog. The zoonym *τσόφλακας* may be genetically related to the term *σοφλάκας* (*soflákas*) of Kotiora (Ordu) in Pontus [32], indicating a potential relationship between the old Greek idioms of Asia Minor, prior to their insularisation by the Turkoman invasions and the mass settlement of the region by mainland Greek-speaking populations [92].

#### 6.3.2. Μπότσκα (bóčka)

*Epirus*: city of Preveza (used by old residents).

*Discussion*. Zoonym of unknown etymology.

#### 6.3.3. Μπουσνιακός (busniakós), μπασιακός (basiakós)

*Thrace*: Avdira. **Μπούσακος (búsakos)**. *Thrace*: Mandra in Xanthi (by Greek-speaking refugees from Serdivan, north-western Asia Minor, opposite Thrace).

*Discussion*. Zoonym of unknown etymology. If the terms *μπουσνιακός* (*busniakós*) and *μπούσακος* (*búsakos*) have a common origin, due to their geographical proximity, they may provide an example of the poorly attested Thraco-Bithynian dialectal family [160]. It should be noted that Avdira is the primary and oldest Greek-speaking settlement in the prefecture of Xanthi, which renders the zoonyms used there of particular linguistic importance. Recording the zoonyms used by speakers of the dialectal varieties of Greek from Eastern Thrace would be of significant importance towards the confirmation of a possible Thraco-Bithynian origin for this term.

#### 6.3.4. Πασπακίνα (paspakína)

*Dodecanese Islands*: Kos (Chora). *Turkey*: Petrumi-Bodrum.

*Discussion*. Informants from Petrumi explained that the term *πασπακίνα* (*paspakína*) was a common zoonym that was used throughout Halicarnassus (modern day Bodrum) prior to the exodus of Greek speakers from the region. It cannot be ascertained whether this zoonym was transferred to the island of Kos through refugees from Petrumi or vice versa. This zoonym is also used to describe an obese and slow-moving person (“he walks slowly like a *πασπακίνα*”).

#### 6.3.5. Κούβακας (kúvakas)

Throughout Chios, single instance in Turkey (Çeşme).

For detailed distribution and additional derivatives, refer to S1 Text (chapter 2.2.4.).

*Discussion*. The zoonym *κούβακας* (*kúvakas*) is found only in Chios. One of our informants, who has a surname derived from *κούβακας* and originates from Çeşme, informed us that his great-grandfather originally received it as a nickname, due to his exceptional swimming skills. The term *κούβακας* may have arrived to Çeşme through the repeated migrations of Chians to Asia Minor, or due to the linguistic and geographical proximity of the two regions.

#### 6.3.6. Κρακάλι (krakáli)

*Italy*: Greek-speakers of Apulia (Castrignano, Calimera, Maglie). **Κορκάλα (korkála)**. *Epirus* (Grammenochoria). **Καρκαρέλι (karkaréli)**. *Ionian Islands*: Zakynthos. **Κάρλακας (kárlakas)**. *Ionian Islands*: Corfu.

*Discussion*. If the zoonyms *κορκάλα* (*korkála*), *κρακάλι* (*krakáli*) and *καρκαρέλι* (*karkaréli*) have a genetic relationship, then they are terms of great antiquity, as Magna Graecia stopped being a part of the Roman Empire in the 12^th^ century. Throughout its distribution, *κρακάλι* and its derivatives typically refer to the frog. It is possible that our informants who refer to toads using the derivatives of this zoonym may have confused it with the frog. However, no other toad zoonym was found among the Greek-speakers of Apulia in this study.

#### 6.3.7. Μπλοκόσα (blokósa)

Thessaloniki (Adam, Petrokerasa).

*Discussion*. Confined to the region of Zangliveri, to the north of Chalkidiki. Derives from the local term *μπλοκός* (= hedge), of uncertain etymology, which refers to the habit of the toad to bury itself along hedges.

#### 6.3.8. Μπάμπακας (bámbakas-bábakas)

North Aegean, exclusively on western Lesbos. **Ξερομπάμπακας (kserobámbakas)**. Pieria.

*Discussion*. Highly local term, deriving from the Greek term *μπάκακας*-*μπάμπακας* [32], of uncertain etymology. The term *μπάκακας* is used elsewhere in Greece to denote the frog [32], but in western Lesbos *μπάμπακας* is used exclusively for the toad, while the frog has different zoonyms (*βαθρακός*, *βατρακός*, *μαθρακός* [32]). Accordingly, in Pieria, the toad is referred to as *ξερομπάμπακας* [from *ξερός* (= dry) + *μπάμπακας* (= frog)] [32].

### 7. Other Vlach zoonyms

#### 7.1. Μπάφα (báfa)

*Macedonia*: Smixi and Perivoli.

*Discussion*. Term of unknown etymology. In Messolonghi, the female flathead grey mullet (Mugil cephalus) is also called *μπάφα*.

#### 7.2. Μπάμπα (bába)

*Thessaly*: in the Vlach villages of Malakasi (Trygona, Pefki, Koutsoufliani).

*Discussion*. A zoonym of unknown etymology, used only to describe unusually large toads, while the term *μπροάτικου* is used exclusively for frogs and small-sized toads. Potentially from Greek *μπάκακας*/*μπάμπακας* [98].

#### 7.3. Μπατσκάβρας (batskávras

*Epirus*: Asprangeloi, Lakka Souli (from Vlach herders).

*Discussion*. Possible combination of *μπατς* (*bats*, perhaps from *μπάτσα* = slap) + *κάβουρας* (*kávouras* = crab, with syncopated -ου). This zoonym is typically used for the scorpion, but in these two villages it also describes the common toad, probably due to the misconception that it spits/ejects poison.

#### 7.4. Φαρφαλιώπα (farfaliópa), φαρφαλιώπε (farfaliópe), φιρφιλιώπα (firfiliópa), χαρχαλιώπα (harhaliópa)

Pockets in Epirus, north-western and Central Macedonia, and Central Greece, exclusively in Arvanitovlach (Rrãmãnji) settlements. **Θαρθαλούπα (tharthalúpa)**, **θουρθουλούπα (thurthulúpa)**. Pockets in Epirus, exclusively used by Vlachs (Armânji).

For detailed distribution, refer to S1 Text (chapter 2.2.5.).

*Discussion*. Loanword from Albanian *thithëlopë* (= cow sucker) (Results 1: subchapter 5.1.). Although *φαρφαλιώπα* and its derivatives clearly represent an Albanian loanword, it is classified here as a Vlach term, as this form is used almost exclusively by the Arvanitovlachs and its distribution mirrors their settlements (Fig 2). Vlachs from the villages of Syrrako and Matsouki also use this zoonym, possibly due to language contact with the Arvanitovlachs in shared winter pastures.

Among Arvanitovlachs, *φαρφαλιώπα* has replaced the original Vlach terms that were occasionally used to describe the toad. For example, in Arvanitovlachs, *μπρουάτικου* (Nižepole, Geroplatanos) is used exclusively for the frog, and *μπρουόσκα*-*μπρόσκα* (Palaiomanina, Stratos in Acarnania, Kria Vrisi in Pella) / *πρόσκα* (Margariti) for the tortoise.

Regarding the dating of the term *φαρφαλιώπα* in Greece, the Arvanitovlachs began to settle permanently outside Albania and Epirus (e.g. Aetolia-Acarnania, Thessaly, Macedonia) from the 18^th^–19^th^ century onwards [161], if not earlier.

### 8. Zoonyms used exclusively by the Pomaks of Western Thrace

#### 8.1. Σταμπόλ (stamból), στάμπολ (stámbol

In all Muslim Slavophone areas of Western Thrace (Greek Thrace).

For detailed distribution, refer to S1 Text (chapter 2.2.6.).

*Discussion*. Zoonym of unknown etymology, although an origin from the toponym Istanbul (= Constantinople) may be used as an augmentative adjective in the region; that is, the toad is the royal frog, because of its girth and size (conceptually similar word to the augmentative zoonyms *βατραχομάνα* and *χοντράδι*). Our informants informed us that in Sminthi and the surrounding villages the term *σταμπόλ* is used exclusively for the toad, while the frog retains its original Slavic zoonym, i.e. *žába*.

#### 8.2. Γιούρτνικ (yúrtnik)

*Thrace*: Sidirokastro Evrou, Mega Dereio = Büyük Derbent.

#### 8.3. Μάμνικ (mámnik)

*Thrace*: Ano Vyrsini, Mega Dereio = Büyük Derbent.

#### 8.4. Παπούτσνικ (papúçnik)

*Thrace*: Aimonio = Valkanova. *Discussion*. Zoonyms of unknown etymology. The suffix -νικ is characteristic of Slavic morphology.

### 9. Gagauz terminology

#### 9.1. Temél kurbağası

*Thrace*: Kleisso.

*Discussion*. Etymologised by the terms *temél* (= foundational, meaning very large) and the Turkish *kurbağası* (= frog), i.e. the monumental frog.

## Results 2: Ethnographic and linguistic data

### 1. Traditions related to udder-sucking of livestock

The belief that the common toad sucks the udders of goats, sheep, and cattle, causing the animals to contract mastitis and die, is prevalent throughout mainland Greece and the Balkans in general. The ethnic and geographical pervasiveness of this belief is absolute, as it is found among all the Greek-speakers, Arvanites, Vlachs, and Slavophones of mainland Greece. This tradition is also common in Albania, where the principal toad zoonym is *thithëlopë* (= cow sucker). According to these beliefs, the toads either attack animals when they are asleep and vulnerable, or they jump and cling to their breasts at night when shepherds are resting. Many also believe that the toad drinks milk directly from the bucket, poisoning humans who consume it, and sometimes jumps into cisterns, rendering the water undrinkable. Many believe that the toad opens slits in the legs of cattle and sucks the blood that gushes out.

The belief of most of our informants in the above traditions is absolute, and more than a few stopped talking to the authors of this study when they showed the slightest doubt on the scientific validity of their claims. Many even swear that they have seen these "parasitic damn frogs" attacking their flocks. It is important to stress that the common toad is a harmless animal whose mouth lacks the musculoskeletal system necessary to perform suction on the teats of livestock. Furthermore, no reptile, bird, or amphibian can digest milk, as they lack the enzyme lactase.

As for the ultimate origin of these traditions and their chronology, one can only speculate. The earliest traditions about animals sucking udders date back to Roman times, if not earlier, and concern birds of the family Caprimulgidae, commonly known as nightjars. Pliny, in his *Natural History*, writes the following: "Those called nightjars resemble a large blackbird and are nocturnal thieves, being blind during the day. They enter the shepherds’ barns and fly onto the goats’ udders to suck their milk, injuring and ruining them, and goats milked in this way gradually become blind" (our translation from Pliny’s Latin text [162]). These traditions are now common throughout Europe, and are especially mirrored in Romance languages (e.g. *chotacabras* in Spanish, *succiacapre* in standard Italian, both meaning goat-sucker).

The beliefs that consider nightjars to be udder-suckers are prevalent throughout Greece, where these birds are known on the mainland as *γιδοβύζι* (goat-tit) and *βυζάστρα* (tit-sucker), while in Crete they are called *αιγοβυζάστρες* (goat-suckers) (our observations). These birds do indeed visit the stables at night, not, of course, to milk the goats, but to feed on the numerous insects that swarm there.

Based on the above, it is possible that the beliefs surrounding the common nightjar may have formed the basis for those of the common toad, as both species visit farms and stables at night. This hypothesis is supported by the fact that in the beliefs about both species, the sucking of the breast brings about the same result–the destruction of the udders and the inability to produce milk, and ultimately the death of the animal–just as Pliny mentioned. In fact, in some regions of Greece, the toad is also called a nightjar (*γιδοβύζι* = goat-tit) (Results 1: subchapter 6.2.1.2.), and it is considered as its volant relative. The superstitions related to the nightjar as a goat-sucker are probably older than the corresponding ones for the toad, as they are found outside the range of this amphibian (e.g. Crete).

The characteristic behaviour of the toad trying to climb the walls of the houses it encounters by putting its legs above its head may have also played an important role in the spread of these beliefs, as an imaginative person could interpret that image as the toad searching for udders to suckle.

A vexing question is why the Cypriots call the skink lizards *Ablepharus budaki* and *Chalcides ocellatus* as *βυζάστρα*-*βυζαστάρι* (= tit-sucker) [99], when the common toad is absent from their island. Although these species are diurnal, when they are near human dwellings, they are usually found under large pieces of metal, wood, or other scraps or debris. Stables and pens provide hiding places as well as plenty of food for these lizards. It is possible that their presence in stables and their lowly and “chthonic” nature as reptiles led Greek Cypriot farmers to assume that they were there to suckle their livestock. The frequent presence of many species of snakes in barns, who are in search of rodents and bird’s eggs, also gave rise to many folk beliefs claiming that serpents visit such places to drink milk from the buckets. These beliefs are widespread throughout Greece’s mainland and islands (Davranoglou, L.-R., personal observation), as well as among the Greek-speakers of South Italy (Vacca, G., personal observation).

Although the folk beliefs that consider the harmless toad as an injurious udder-sucker of livestock are lost in the depths of time, their presence among the Albanians, the Vlachs, and the Greeks indicate that the Balkan region was crucial for the development or at least the dissemination of these traditions. It is worth mentioning here that in the regions that retain zoonyms from the archaic terms *φρῦνος* and *φροῦνος* (Andros, Euboea, Pontus), the beliefs that consider this species as an udder-sucker are entirely absent. In fact, in Andros (among both Greek-speakers and Arvanites), in Euboea (Kymi and Karystos), as well as Muslim Pontic speakers (Sitaridou, personal observation), this animal is particularly loved, and it is considered to be the farmer’s friend as it feeds on pests. In Andros, some refer to it as the “animal of the Virgin Mary” (*ζώο της Παναγίας*). Indeed, in ancient Greece, the common toad was probably held in some regard, as many personal names were based on it (Results 1: subchapter 1), a striking example being the beautiful courtesan Phryne, nicknamed as such by the Athenians because of the bronze-brown colour of her skin [65].

Therefore, the association of the common toad with udder-sucking might represent a later phenomenon, probably of the Roman period. After all, the Romans already had the associated tradition concerning the nightjar [162], and in general held negative perceptions about the toad [163]. The present study also proposed that the zoonym *βούζα* (“titter”, or tit-sucker) was coined precisely in the period when Roman influence in the Balkans was significant (1^st^–6^th^ centuries AD) [48, 49]. These beliefs, together with the change of attitudes towards amphibians and reptiles, which in early Christianity came to be seen as demonic beings, must have been responsible for the negative image that the toad acquired, and perhaps for the disappearance of the “pagan” personal names based on *φρῦν*- and *φροῦν*-.

### 2. Confusion between toad zoonyms with those of the tortoise in Vlach and its linguistic significance

Our informants of Vlach origin often disagreed about whether the term *bruásca* denoted the tortoise or the toad. In particular, several conceptual differences were discovered, which are summarized in Table 1.

**Discussion.** The conceptual confusion between the frog or toad and the tortoise is frequently found in other languages as well. In Turkish, the tortoise is known as *tısbağa* [stone-frog: (*daş* (= stone) +‎ *bağa* (frog or toad))], or *kaplumbağa* [covered frog or toad: *kaplı* (cover) +‎ *bağa*] [164]. In German, the tortoise is called *Schildkröte* [shielded toad: *Schild* (= shield) + *Kröte* (= toad)], while in Romanian it is called *broască-țestoasă* [frog with shell: *broască* (= frog) + *țestoasă* (= shell)] [164]. Taking the above information into account, together with our data from the Vlachs, it is clear that the interpretation of the tortoise as a shelled/covered/saddled toad, or the latter as a homeless or naked turtle, evolved independently in many different peoples. Because of the toad’s plump and rounded appearance, dark brown colour, and short limbs, it is not difficult for one to imagine it as the homeless or naked relative of the tortoise.

These conceptual differences have caused particular confusion among speakers of Vlach, with different villages ardently arguing over whether *bruásca* refers to the tortoise or the toad. This is also due to the fact that differences in naming are often accompanied by particular beliefs. For example, in Zagori, according to a local belief among Greek-speakers (probably influenced by Vlachs), the toad originally had a shell, like other tortoises, but because it fell into Communion, God cursed it, causing the toad to lose its shell and roam naked for eternity.

The semantic shifts between toad and tortoise (and vice versa) in Vlach may have a particular complex history, and are inextricably linked to proto-Albanian, a language that may have been spoken by the Vlachs prior to their Romanisation [5–12]. Although the term *bruásca* in its Hellenised form *μπράσκα* refers exclusively to the toad, it has been shown here that in many Vlach populations it denotes the tortoise (Table 1), despite the fact that it originates from the vulgar Latin term *broscus* (= frog), which in turn may derive from the ancient Greek *βροτάχος* [128, 129]. The related Albanian term *breshkë* (Hellenised as *μπρέσκλα*-*μπρέσκα*), although also stemming from *broscus*, refers to the tortoise and not to the frog, the original meaning of the loan. These semantic differences may be explained if it is hypothesized that prior to loaning the term *broscus* from Latin, the proto-Albanian and proto-Vlach lexicon had already incorporated a term for the frog directly from the ancient Greek *βροτάχος*, which gave Vlach *bruáticu* and Albanian *bretkosë*, both of which mean the frog. Therefore, proto-Albanian and proto-Vlach loaned frog zoonyms twice in their historical development, once directly from Greek (*βρόταχος*) and once from Latin (*broscus*).

**Fig 13:**
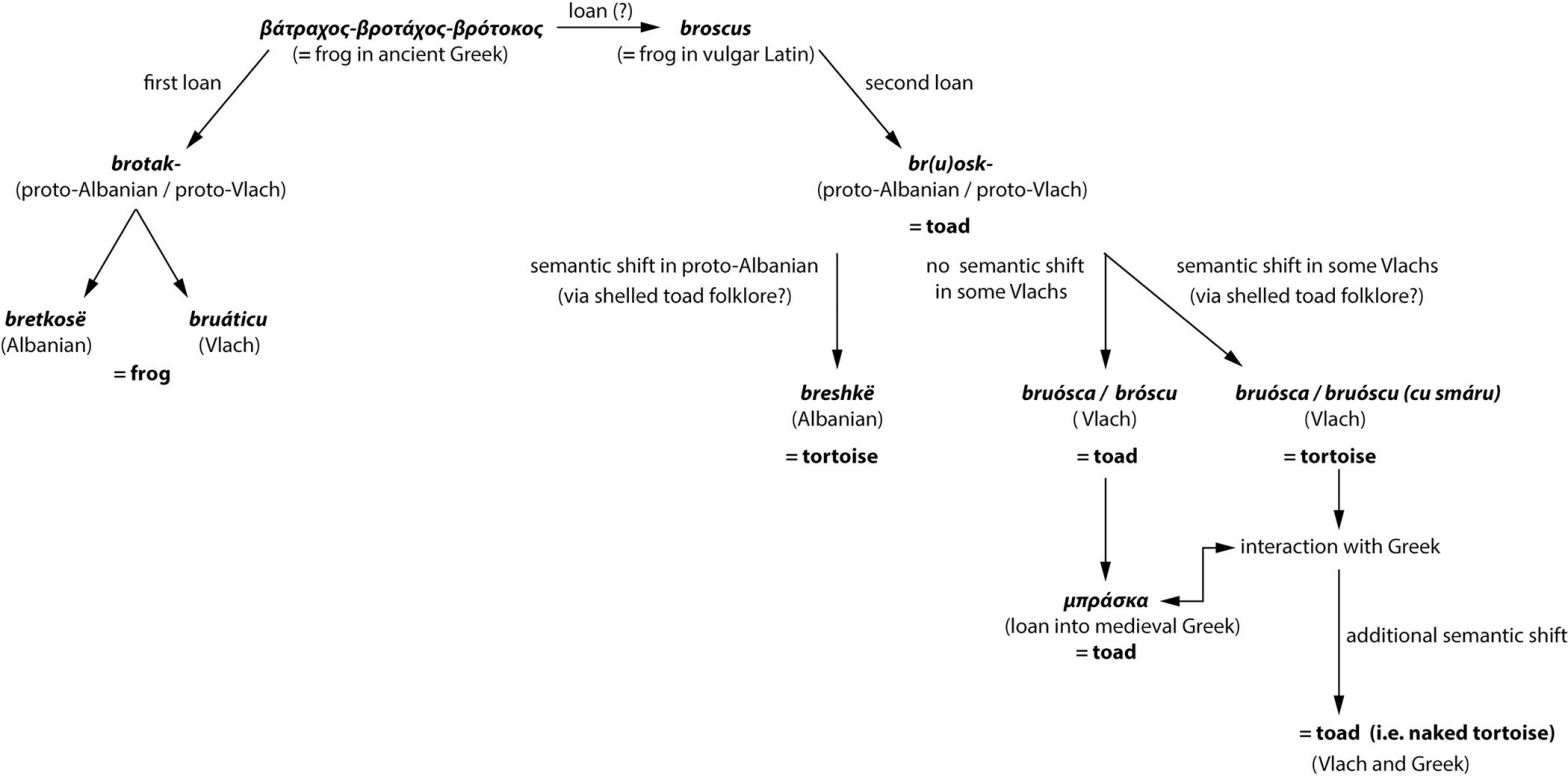
Hypothetical reconstruction of the linguistic and semantic evolution of the ancient Greek zoonym *βροτάχος* and vulgar Latin *broscus* in proto-Albanian and proto-Vlach.

The hypothetical double introduction of frog zoonyms in proto-Albanian and proto-Vlach may have led to several semantic shifts which are mentioned below: (i) when the Greek term *βροτάχος* first entered proto-Albanian / proto-Vlach as *brotak*-, its meaning stayed the same (= frog; evolved into *bruáticu* in Vlach and into *bretkosë* in Albanian); (ii) when *broscus* was loaned from Latin into proto-Albanian and proto-Vlach, its meaning changed to refer to the toad (*bruósca*/*bruásca*-*breshkë*) as zoonyms for the frog were already available (*bruáticu*, *bretkosë*); (iii) perhaps through folkloric mediation, the tortoise was independently re-interpreted as a “shelled toad” in all Albanian populations and also in a subset of Vlachs; (iii) in those Vlach populations who used *bruásca* to refer to the tortoise, an additional semantic shift took place, where the toad was re-interpreted as a naked tortoise, and was therefore given the zoonym *bruásca* once more; this last shift may have been achieved as a consequence of the Vlachs’ interactions with Greek-speakers who used *μπράσκα* almost exclusively for the toad. A summary of this hypothesis is provided in Fig 13.

The significant semantic differences of the zoonym *bruósca*/*bruásca* in Vlach are likely a consequence of the Vlachs’ scattered and highly mobile populations, which prevented dialect levelling from taking place. On the other hand, the Albanians, although originally nomadic, may have formed settled agricultural populations earlier than the Vlachs, which enabled dialect levelling to occur, with the zoonym *thithëlopë* arising as the dominant term for the toad.

It should also be noted that the interpretation of the Greek zoonym *βροτάχος* as a Greek loan into proto-Albanian (and proto-Vlach) conforms with the current understanding of the evolution of the proto-Albanian language. Indeed, the zoonym *bretkosë* can be satisfactorily interpreted as the result of the following series of transformations:

*βροτάχος* > *br****o****tak*- > *br****uo****tak* > *br****ue****tak* > *br****e****tk* > *br****e****tkosë*

Indeed, the transformation *ō>uo>ue>e is a typical proto-Albanian development (e.g. Latin *h****o****ra* > Albanian *h****e****rë*; Latin *p****o****mum* > Albanian *p****e****më*) [9, 120].

A similar process may have taken place with the loan of *broscus* into proto-Albanian that resulted in the zoonym *breshkë*:

*broscus* > *br****(****u)o****sc****us* > *bre****sc****us* > *bre****shk****ë*

The transformation **sk>shk** is typical of how proto-Albanian treated Latin loans (and non-native terms more broadly) e.g. Latin ***sc****ala* > Albanian ***shk****allë*; Latin *pi****sc****is* > Albanian *pe****shk***; Latin *mu****sc****ellarius* > Albanian *mu****shk****ëllyer* [120].

Furthermore, the frequent transformation *ō>oa/ua in *br****o****scus* > *br****uó****sca/br****uá****sca* in Vlach is also typical for this language e.g. Latin *n****o****ctem* > Vlach *n****oa****pte*/*n****oa****pti*; Latin *coxa* > Vlach *c****oa****psă*.

### 3. The toad as a descriptive term for humans and animals

Due to the toad’s perceived ugliness, and also because of its obese-like appearance, toad zoonyms are often used as nicknames. Almost all of the zoonyms of this amphibian are also found as surnames (which are not mentioned here for privacy reasons). Moreover, the zoonyms of the toad are used either as pejorative terms to describe a woman’s appearance (e.g. "she’s ugly as a *βούζα*/*μπράσκα*/*ζιάμπα*; she looks terrible, she’s like a *ζιάμπα* "), but also as a swear word between women ("you terrible woman, you’re a *μπράσκα* "). Additionally, throughout Aetolia, the alleged envenomating behaviour of the toad is used as a curse (“may the *mšáka*-*bžáka* sting you”).

Furthermore, the size and plump appearance of the toad has inspired a number of metaphors and similes, especially those related to heavy eating or sudden weight gain (e.g. "I ate a lot and I swelled up like a *βούζα*; I got fat and I’ve gained a *βουζαρίκα*; I made a *βούζα*” i.e. I got a belly).

The zoonyms of *Bufo bufo* are also used to describe negative emotions, probably due to an anthropomorphism of the apparent frowning expression of the species, e.g. he/she is sulking like a *βούζα*/*μπράσκα*/*ζιάμπα*.

It is also interesting to note that in the city of Volos and in many other places, the toad zoonym *μπράσκα* has been transferred to the common monkfish [*Lophius piscatorius*–otherwise known as *πεσκανδρίτσα (peskandrítsa)*], due to its superficial similarity to the amphibian (Davranoglou & Embirikos, personal observation). The same process has taken place for the toadfish (family Batrachoididae), which has been named after the amphibian in English, Catalan (*gripau*), and Spanish (*pez sapo*).

### 4. Zoonyms of the green toad (*Bufotes viridis*)

The second member of the family Bufonidae in Greece is the green toad (*Bufotes viridis*), which lacks any zoonym that is specifically dedicated to this species in any region of the country. Across its distribution in Greece, the zoonyms of the frog are assigned to the green toad. The following indicative examples are provided: *αφορδακός*/*μπαρδακός* (Crete), *βόθρακος*/*βόδρακος*/*βορτακούδιν* (Cyprus), *βαθρακλάς* (Mani), *φαρδακλός* (Rhodes), *φουρδακλάς*/*φορδακλάς*/*φορτακλός* (Leukada), *φαρδακάς* (Tsakonia), *μπάκακας* (Attica, Phocis, Thrace, Pelasgia, Pelion, Granitsa of Ioannina, Meliki Amfilochias, Ilia), *μπάμπακας* (Kolindros in Pieria).

The zoogeography of *Bufotes viridis* may also provide useful information about the way this animal was named in ancient Greece. The green toad is more drought resistant than the common toad and is found in areas where the latter is absent (e.g. Cyclades, Dodecanese) [99]. In these areas the green toad is always masked by the zoonyms of the frog, as shown above. One would expect that if the Greeks of classical times referred to this species as *φρῦνος*, the zoonym would have survived on these islands. However, only in Andros does the zoonym *φρῦνος* survive in the form of *φουρνός*, where it is used exclusively for the common toad. If one also considers that the term *φρῦνος* probably derives from the proto-Indo-European term *bʰerH- (= brownish), which seems so as it was given as a nickname to the courtesan Phryne to describe the dark colour of her skin [65], then it is reasonable to assume that *Bufotes viridis*, which is green, was never called this zoonym by the ancient Greeks, as its meaning would not apply to this species. Therefore, the application of frog zoonyms to *Bufotes viridis* must be particularly old.

### 5. The complex social use of toad zoonyms in Greece

Although the official Greek name of the common toad is the ancient Greek *φρῦνος*, the present research has shown that this zoonym is entirely absent in the countryside, where the names of this species are very diverse–more so than in any other region in Europe (at least), to the authors’ knowledge. The zoonym *φρῦνος* is used solely by scientists and some members of the urban class who are interested in natural history. The artificial resurrection of a zoonym that was forgotten for almost 2.000 years may explain why it has never gained appeal among the majority of the country’s population. In fact, of the 7.700 informants of this study, only 17 used the zoonym *φρῦνος*, and these were individuals who were part of natural-history enthusiast groups where the term is known. The urban nature of the term *φρῦνος* has the effect of obscuring the “lesser names” of the toad in Greece. This phenomenon originates from the deeply rustic character of the toad’s zoonyms, and many villagers, upon mentioning a toad zoonym to urbanised individuals, immediately realise that they are speaking of something that is not part of the official version of Standard Modern Greek. That is, by merely referring to the toad with a zoonym other than *φρύνος*, one reveals their rural origins. One can therefore infer that the basic characteristic of the Greek communicative sphere is that idiomatic terms, which are invariably of rural nature, can be tolerated in the restricted environment of a village or a small town, but may be considered as improper in the broader public sphere. However, even within rural populations, the most widely used toad zoonyms obscure the minor ones. For example, an informant said that "*X* is the *correct* name of the animal, it is called *Y* only by the *βλάχοι* (= Vlachs, in the pejorative sense of the term, meaning an uneducated rural person)", while some Euboeans who knew the toad as *μπράσκα* corrected their neighbours when they used the “incorrect” term *φουρνία*.

Based on the above, in Greece, the toad could be classified as a “minority” animal in the Deleuzeian sense of the *devenir minoritaire* (i.e. “becoming a minority”) [165], which is a process that is not related to numbers, nor does it have a political sense, but refers to concepts that are radically different from the dominant ones. In the case of the toad, its zoonyms are masked (i.e. acquire “minority” status) in Standard Modern Greek by the term frog, or even the academic term *φρύνος*. However, the toad becomes a “minority” animal for additional reasons, which are explained below.

The toad is a “minority species” in the Greek world, as it has remained almost completely unexplored from an anthropological, ethnozoological, and historical point of view, and for this reason many of its zoonyms and the beliefs that accompany them are recorded for the first time in this study. This is due to the fact that research in the above-mentioned fields primarily examines the interactions of humans with species that play an important role in the agricultural economy. The toad, however, is of no economic interest, as its relationship with humans is limited to (erroneous) folk beliefs about its harmfulness to livestock and humans (Results 2: subchapter 1).

Another reason why the common toad is a “minority” animal relates to the fact that it is completely obscured in the modern Greek written tradition, as it is usually concealed by the frog. While in northern and western Europe it is the toad that occupies a central role in fairy tales and folklore (e.g. a toad is kissed by the king’s daughter in a Grimm fairy tale, and a toad was supposedly dressed and nurtured by witches in the Basque Country [29]), in Greek translations of western European fairy tales the toad is invariably replaced by the zoonym *βάτραχος*, which means frog in Standard Modern Greek.

The principal reason why the toad is a “minority” animal in Greece, is that the majority of its zoonyms reflect the historical evolution of the varieties of the Greek language, and how they were in contact with Slavic, Vlach, and Arvanitika (Figs 2, 8A and 8B). Therefore, the toad’s zoonyms provide evidence for the linguistic and demographic continuities and discontinuities of Greece. Consequently, the toad is a particularly useful linguistic tool, especially when considering that mainland Greece lacks a dialect atlas [38], which leaves the distribution and historical evolution of the spoken varieties of Greek and other languages inadequately documented. This is particularly problematic for linguistic research in the country, as many of these dialects and languages are vanishing at an alarming pace.

Although the toad’s zoonyms could have been used as an isogloss, this was not possible, due to the animal’s absence from significant parts of the Greek-speaking area (e.g. most islands).

### 6. Concluding remarks

The collective imaginary that perceives the toad as a chthonic animal that is dangerous to both livestock and humans undoubtedly played a crucial role in the transmission and preservation of its zoonyms throughout Greece. It is not difficult to imagine the populations of the countryside–from farmers to transhumant shepherds–recounting their risky (in their imagination) encounters with this animal, comparing their beliefs, but also its multiple (and often intentionally comical) names. One can also imagine mothers teaching their children to avoid this toxic animal, using the traditional names of their homeland, which are not included in the official language they learned at school.

Having the abovementioned social background in mind, the strongly local character of the toad’s zoonyms is unsurprising. Indeed, this research highlights the importance of zoonyms as substrate terms, especially those used for animals of minimal economic importance, such as reptiles and amphibians, which often escape the attention of the dominant languages, and therefore resist linguistic substitution. It has been shown here that the examination of a single species, the common toad, may shed light on historical and demographic processes that took place throughout Greece from antiquity to the present day, and may be applied for other ethnolinguistic groups as well.

Indeed, the findings presented here provide additional evidence for processes that shaped the evolution of the Greek language itself. Although the country’s standard linguistic literature [166] considers the Modern Greek language as a direct descendant of the Hellenistic koine, the present study adds further support to hypotheses suggesting that a more complex scenario may have taken place. Previous studies have proposed that the spoken varieties of the Greek language evolved through the mixture of various dialectal varieties of Hellenistic koine, and other languages [**koineization**], mainly in Constantinople, but also in areas of linguistic contact [96, 100, 102–104]. These influences varied from region to region, shaping the dialectal diversity of Greek that is observed today. The data on the humble toad presented here are also consistent with these observations, as today’s dialectal varieties of Greek retain zoonyms that can be traced to various historical periods–from classical (*φουρνός*, *φουρνόν*, *φουρνία*, *φουρνιά*) and late antiquity (*βούζα*, *φουσάκλα*, *σακούδα*, *σακκούτα*), to the Migration Period (*ζάμπα*, *μπράσκα*) and the contraction of the Roman Empire, the late Middle Ages (*ασκουβάζα*, *θυθλιώπα*, *μπζάκα*, *μπάτσιακας*, *μπουσνιακός*), the huge expansion of transhumant pastoralism in the 17^th^ century (*μπράσκα*), and developments of more recent times (e.g. *φιρφιλιώπα*, *χοντράδι*, *βατραχομάνα*, *μασταράς*).

Overall, this research stresses the fundamental importance of exploring and preserving linguistic diversity on a global scale, as it represents an exceptional tool for understanding humanity’s shared history, to investigate the similarities and differences on how people interacted with their surroundings, and to preserve different ways of meaning that do not conform to the 21^st^ century Western perspective. In a European context, this is particularly pertinent to Greece, as it is the continent’s only country that lacks a dialectal atlas. To this end, the authors of this study have begun recording all of the country’s reptile and amphibian zoonyms, where both field research and online interviews are crucial (Davranoglou, Embirikos & Strachinis, in preparation). By showcasing the utility of the toad’s zoonyms in anthropological, ethnozoological, historical, and linguistic studies, it is hoped that researchers in these fields will further explore the potential of zoonyms as substrate terms in their group(s) of study.

## Supporting information

S1 Text Study methodology and detailed list of variants of toad zoonyms and their distribution. (DOCX)

S1 Table Altitudinal data used for Kruskal-Wallis test. (CSV)
