# Supplementary material for: Toad zoonyms mirror the linguistic and demographic history of Greece
Source: PLoS One. 2023 Mar 29;18(3):e0283136. doi: 10.1371/journal.pone.0283136 (PMC10057758; doi:10.1371/journal.pone.0283136)
Supplement: S1 Text — (DOCX) [file pone.0283136.s001.docx]

**Supplementary Information**

**Contents:**

**1. Methodology**

**1.1. Rationale for field data sampling locations…………………………………………………2**

**1.2. Data collection in the field………………………………………………………………...3-5**

**1.3. Methodology for online survey……………………………………………………………5-9**

**2. Geographical distribution of zoonyms at village level.**

**2.1.1. Φουρνία** (**furnía) and derivatives………………………………………………………..10**

**2.1.2. Βούζα (vúza)** **and derivatives…………………………………………………………10-11**

**2.1.3. Μπζάκα (bžáka) and derivatives……………………………………………………..11-12**

**2.1.4. Ζάμπα (žába) and derivatives………………………………………………………...12-13**

**2.1.5. In Pomak………………………………………………………………………………13-14**

**2.1.6. In Slavophones (“ντόπια”, Slavomacedonian, Makedonski).…………….…………….14**

**2.1.7. Μπάτζιακας (bátziakas)** **and derivatives…………………………………………..……14**

**2.1.8. Μπράσκα (brásca) and derivatives…………………………………………………..15-17**

**2.1.9. Θιθιλιώπα (thithiliópa) and derivatives…………………………………………………17**

**2.2.1. Μπρέσκλα (bréskla) and derivatives…………………………………………………….17**

**2.2.2. Μπερτικοσα (bertikósa) and derivatives………………………………………………..18**

**2.2.3. Μπρασκαφούτα (brascafúta) and derivatives…………..……….……………………..18**

**2.2.4. Κούβακας (kúvakas) and derivatives ……….……………………………………….18-19**

**2.2.5. Φαρφαλιώπα (farfaliópa) and derivatives………………………………………………19**

**2.2.6. Σταμπόλ (stamból), στάμπολ (stámbol)…………………………………………………19**

**3. References…………………………………………………………………………………….19**

**1. Methodology.**

**1.1. Rationale for field data sampling locations.**

With few exceptions, large urban centers (e.g. Athens, Thessaloniki, Chalkida) were not sampled, not only to avoid collecting information from individuals of unknown origin, but also for the practical reason that most urbanised Greeks cannot distinguish the common toad from the frog. This study did not interview Roma/Romani individuals, as rural populations of this ethnolinguistic group tend to use the dominant zoonyms employed by local Greek-speakers. Greek Romaniote and Sephardic Jews were also not interviewed, as non-urbanised individuals who would be familiar with toads could not be located by the authors of this study. This study also did not interview Turkish individuals of the Muslim minority of Western Thrace. Future research should aim to record zoonyms from the abovementioned groups that were not sampled herein.

Also, the zoogeography of the species played an important role in sampling choice, as *Bufo bufo* is not found in the Cyclades (except Andros), the Dodecanese (except Kos), Crete, Cyprus, and most of the Aegean islands (e.g. Aegina, Cythera, Salamina, Hydra, Spetses, Ithaca), with the exception of Samothraki, Thasos, Chios, and Lesbos. Consequently, individuals from those areas were *Bufo bufo* is not found were not interviewed.

**1.2. Data collection in the field.**

All field data collected in this study originate from both authors’ casual conversations with residents of the Greek countryside, mainly with those involved in the agricultural and livestock sectors, who have more contact with nature and are more familiar with local animal names. The first author (Davranoglou, L.-R.) collected field data mainly from Arcadia, Epirus, Corfu, Mani, and Messinia during zoological surveys, where he conversed with locals and was frequently hosted by them and their families. The second author (Embirikos, L.), who collected the majority of the field data, conducted ethnomusicological and historical research across Greece for more than 35 years as a research associate of the Musical Folklore Archive of the Centre for Asia Minor Studies in Athens, Greece, with the task of recording the country’s music, traditions, idioms, dialects, languages and historical data. In this way, this author interacted with thousands of individuals across Greece, including the primary non-Greek-speaking populations of the country (e.g. Slavs, Vlachs, Arvanites) and speakers of divergent forms of modern Greek (Pontians, Tsakonians, etc.), with whom he discussed about their music, their language, their folklore, and traditions.

During those informal discussions, this author obtained **verbal consent** from the participants in the following ways:

**1. The scope of the research goals was made clear.** The author, either through introduction by other locals, or by himself, explained to the locals the goals of their research (i.e. the preservation and recording Greece’s music, language, folklore, and recent history). After the author realised the potential importance of toad zoonyms in the languages of Greece, he informed the locals about his interest in recording the names of the toad, with the ultimate purpose of using them in a linguistic-anthropological-ethnographic-historical study. The reason why written consent was not sought rests entirely upon the fact that all discussions took place in a spontaneous manner, during chance meetings in communal spaces or in nature. Written consent could have also made the locals suspicious about the researcher’s intentions, with the result of hindering an otherwise informal discussion.

**2. The benefits and use of the data was made clear**, as the author explained that he is recording the music, languages, and traditions of Greece for academic purposes, which would be disseminated in the form of books, publications, and audio recordings (the latter is not applicable for this study) in Greek and in international languages.

**3. Participation was entirely voluntary**, as many of these discussions took place spontaneously and in an informal fashion (e.g. while discussing with elders in the village café, in family gatherings, and chance meetings with farmers and shepherds in nature). The participants could change the topic or leave the conversation whenever they wished.

**4. No identifiable characteristics of the participants were retained**, as the study took place during informal discussions and chance meetings. The author would then keep the zoonyms he heard in his memory, until writing them down a few hours or days later. Due to the spontaneous and informal nature of the meetings, the author did not ask for the name or any other personal details of any of the participants, with the exception of close personal friends.

**5. No reasonable risks or discomforts to the participants could be identified**, as the study’s question merely concerned the common name of an inoffensive species of amphibian. All participants were particularly enthusiastic to share the local name of this animal, as it reminded them of their younger years, and many found the study’s question amusing, due to the often comical traditions/properties that are associated in folk tradition with the common European toad.

**6. Information about time and compensation was not required**, as the time spent answering the question was decided entirely by the participants. No compensation was given, as these records took place during informal discussions. **A timeline for the publication of results was also not mentioned**, as the author could not estimate how long it would take to satisfactorily document the zoonyms of the common toad across Greece, especially in the pre-digital era of the late 1980s and early 1990s when the research took place.

**7. Who could participate in this study?** Anyone present in the village, irrespective of gender, ethnolinguistic affiliation, or religion, participated in the author’s discussions. No one was excluded from what was a friendly and often communal conversation.

**8. All participants were over 18 years of age**. In fact, the vast majority of the people who participated in the field were elderly, as they retained the folk memory of their area, and had a deeper contact with nature, unlike their now largely urbanised descendants, who generally cannot identify the common European toad. In fact, as many of the participants were of advanced age already in the 1990s when these discussions took place, it is assumed that a large number of them are now deceased.

The corresponding author (Davranoglou, L.-R) submitted a query on whether the collected field data required ethical review to the Social Sciences and Humanities Interdivisional Research Ethics Committee (SSH IDREC) of the University of Oxford. The response from the SSH IDREC explicitly stated and confirmed that previously collected field data were not subject to ethical review, as the dataset was transferred to the University of Oxford in an anonymised format (i.e. no personal information was available) and data collection took place prior to the commencement of the corresponding author’s Fellowship at the University. The same query was submitted to the appropriate team of the author’s division (Research and Impact Team of the University of Oxford’s Gardens, Libraries and Museums), whose members also confirmed that field data did not require ethical review for the same reasons specified by the SSH IDREC.

**1.3. Methodology for online survey.**

In our *in silico* era, social media and citizen participation in all types of scientific research (citizen science) are becoming increasingly important. Although this approach has gained popularity in the sciences, the same cannot be said for research studying linguistics, folklore, and oral traditions. The internet provides an almost infinite source of information, brings the researcher closer to a large number of people in a short time frame, and allows for the collection of data from across the globe. One of the aims of our research is to highlight the importance of the internet as an additional aid in the researcher’s toolbox, without underestimating the undeniable and primary importance of collecting data from the field.

All online data collection was undertaken with the permission of SSH IDREC (permit number R81032/RE001). The online citizen science volunteers participated through Facebook groups dedicated to Greek nature, culture, and the dialects-languages of a particular region or village, whose members share photographs of local animals, architecture and customs, or ask how a particular animal/plant/expression/agricultural tool, etc. is called in their local idiom, dialect, or language.

The author responsible for online data collection (Davranoglou, L.-R.) sought permission of the administrator(s) of each group to conduct this research. Once each group’s administrator(s) approved the author’s request, the author then wrote a public post in informal Standard Modern Greek, where he posted the following question, seeking the **informed written consent** of the participants (text in parentheses included here only for explanatory purposes):

"*Hello, I am X* (full name shown on Facebook profile) *and I am* *conducting research on the common names of animals in Greece. Would you like to let me know how this animal is called in your village* (photo of animal shown)*, and where in Greece you are from?*

*Your responses will be recorded anonymously and will be used in a linguistic study that examines the dialects, idioms, and languages of Greece. The purpose of my research is to try to map the linguistic diversity of Greece, understand its origins, and contribute towards its preservation for the generations to come through publications in international and Greek journals.*

*If you agree with the above, I would love to hear how you call this critter in your area. The posts will be subsequently deleted to protect your anonymity.*

*Thank you for your help!*"

The authors’ question was always accompanied by a photograph of the common toad, often together with photographs of the green toad (*Bufotes viridis*) and frogs inhabiting the region of study (*Pelophylax* spp., *Rana* spp.). The origin of each informant and the location in which they grew up were ascertained. Furthermore, only those zoonyms that were confirmed to occur in a given location by more than two unrelated individuals were used in this study. Each informant's responses were compared with the authors’ field observations to identify any "anomalies" in the distribution of each zoonym.

The question used for online data collection fulfills the following criteria of informed consent:

**1. Answering the study’s question was entirely voluntary**, i.e. only those members who were interested in answering to the author’s post did so. No member was pressured or coerced to answer the question, as the Facebook post **was not** directed towards any specific person, but was part of the group's feed, among many other members’ posts (some of these Facebook groups have over 10.000 members). The author’s question **was not** part of a pinned post (i.e. a post that is displayed permanently on the first page of a Facebook group), but after a few hours it was “buried” by newer posts of other members. Furthermore, the degree of information provided (village, animal name, associated folklore, etc.) was left to the discretion of the participants themselves, not only to ensure their privacy, but also to prevent biasing the results.

**2.** **The research purpose is stated clearly** (to document a word in the dialects and languages of Greece and to linguistically and historically analyse their origins). No detailed explanation of the research methodology was required, as it merely consisted of the consultation of etymological dictionaries of the idioms, dialects, and languages of Greece, and the associated literature in linguistics, ethnography, and history of the region. The only statistical method employed was a Kruskal-Wallis test, which was used to test the relationship between a particular zoonym and altitude. The duration of the online part of the study was not mentioned, as it started as a part-time project of one of the co-authors that lacked a fixed deadline (Davranoglou, L.R.).

**3. The benefits of the study are clear**, that is, the documentation and preservation of local folk names and beliefs for the generations to come. The study’s results (which are open access) will be shared in all the Facebook groups that host the participants of this study. The participants’ responses will not and cannot be used for financial, clinical, or political goals.

**4. The anonymity of the participants was ensured. The study’s posts on the Facebook groups were subsequently deleted**, so that the responses and usernames of the participants **could not be traced. No identifying information of the participants was stored or disseminated in any way** (e.g. via screenshots, excel files containing personal names, sharing of the latter through personal messages or emails, etc.) Only the participants and group administrators who **specifically asked** for their person or Facebook group to be named are mentioned in the Acknowledgements of the study.

The only data that were stored were the zoonyms and associated beliefs found in a particular village, **without the names of the participants or any other personal information**. A hypothetical example is provided below:

List of toad zoonyms:

*μπράσκα*, Sarakatsani village (participants said term is used only by the elderly)

*βούζα*, Thiva (broadly used, confirmed by 13 participants)

*βούζα*, Corinthia (exact location not provided by participant)

*μπράσκα*, Laconia (considered to be venomous, confirmed by 30 local participants)

**5. No reasonable risks or discomforts to the participants could be identified**, as the study’s question concerned the common name of an inoffensive species of amphibian, i.e. an uncontroversial topic that does not touch upon or affect a participant’s sensitivities. Furthermore, as the study’s posts were deleted after 48 hours, the anonymity of the participants was ensured. It should be noted that all participants were particularly eager to share the local name of this animal, and many found the study’s question amusing, due to the often comical traditions/properties that are associated with the common European toad.

**6. Information about time and compensation was not required**, as the Facebook members decided whether they would answer the study’s question, and how much time they would spend to type their answer. Most answers to the study’s question had the following structure, which is estimated not to have required more than a few seconds to type (hypothetical example):

Post 1: *Askuvaza* (the zoonym in question)*, common in Argolida.*

Post 2*: Brasca, I heard it in X and Y villages of Pelion.*

As no monetary compensation was offered to the participants, the study’s question did not include such a section.

**7. Who could participate in this study?** From the study question it was clear that the target audience are people from all ethnolinguistic backgrounds of Greece, irrespective of gender, religion, or language. Nearly 100% of the participants were Greek citizens, as the Facebook groups where the study question was posted concern Greek matters, whose members write almost exclusively in the idioms and languages of the country (Standard Modern Greek, Tsakonian, Vlach, Arvanite, etc.). As some of the speakers of the aforementioned dialects and languages also live outside the borders of the Hellenic Republic, a small number of responses (9 people) from members from neighbouring countries was also received. Their answers were also included in the study.

**8. All of the study’s participants were over 18 years of age**, as this was a prerequisite of many of the Facebook groups that hosted them. Furthermore, a very considerable portion of the study’s participants were elderly, as they retained the old zoonyms of their region, which are unknown or rarely mentioned among the younger generations.

**2. Geographical distribution of zoonyms at village level.**

**2.1.1. Φουρνία** (**furnía) and derivatives.**

**Φουρνία** (**furnía).** *Central Greece*: Euboea (Androniani, Kimi, Vitala, Gaia, Enoria, Kalimerani, Potamia, Pyrgos Kimis, Kourouni, Konistres, Oxylithos, Gavalas of Avlonari, Agios Georgios Avlonariou). **Σκαφουρνία (skafurnía)**. *Central Greece*: Euboea (Gaia). **Φουρνιά (furniá).** *Central Greece*: Euboea (Agios Ioannis Aliveriou, Agios Loukas Aliveriou, Aliveri, Karystos). **Φουρνός (furnós)**. *Central Greece*: Euboea (Karystos). *Cyclades*: Central and south Andros [grass snakes (*Natrix natrix*) which feed on toads are called *φουρνοφάϊσες* (= toad eaters)]. **Φούρνος (fúrnos)**. *Turkey*: Pontus (Sanda = Dumanli) [1]. **Φουρνόν (****furnón)**. *Turkey*: used by all speakers of Pontic Greek: Argyropolis = Gümüşhane, Kerasounta = Giresun, Samsounta = Samsun (only in settlements of Argyropolitans), Trapezounta = Trabzon, today found in all of the Black Sea Romeyka-speaking villages [Tonya villages, Matsouka = Maçka, Sarahos = Uzungöl, Surmena = Sürmene, Kandahor = Çaykara, Anasta (Sitaridou, I., personal communication)]. **Φρούνος (frúnos)**. *Turkey*: Pontus (Chaldia) [1].

**2.1.2. Βούζα (vúza)** **and derivatives.**

**Βούζα (vúza)**. *Macedonia*: Pylaia in Thessaloniki. *North Aegean*: Samos (Pagonda). *Peloponnese*: Achaia (widespread e.g. Aigio, generally in Aigialeia, Mesino, Aigeira Monastery, Lakkopetra, Lakkomata Erymanthou, Patras, Spodiana), Arcadia (Vlacherna, Dimitsana), Ilia [areas from Kyllini to Andravida e.g. Amaliada, Arini, Dafniotissa, Lechaina, Kardamas, Kourtesi, Peristeri, Varda, Manolada, Melissa of Lechaina, Myrtia, Chavari, Psari (from Arvanites)], Corinthia (the most common and widespread name throughout the prefecture, e.g. Ano Karteri, Assos, Velo, general area of Vocha, Vrachati (also from Arvanites), Drosopigi, Examilia, Gioza, Driza, Kalavryta, Kaliani, Kastania, Kato Diminio, Kefalari, Kionia, Kyllini Corinthias, Kiras Vrysi (Arvanites), Klimenti, Corinthos, Laliotis, Lafka, Lechaio, Lygia, Nafplio (Arvanites), Perachora (Arvanites), Stymfalia, Manna, Megalos Valtos, Mosia, Bouzi, Xylokastro and surrounding villages, Stylia, Zevgolatio). *Central Greece*: Aetolia-Acarnania (Karpenissi and surrounding villages), Boeotia [Agios Georgios, Agia Paraskevi, Agios Spiridon, Drachmani-Elateia, Davleia, Distomo, Evangelistria (Arvanites), Thebes, Kamena Vourla, Kalapodi, Kaloskopi, Kastro, Kokkino (Arvanites), Lefktra (Arvanites), Leivadia, Agia Anna of Leivadia (Arvanites), villages of Mt Parnassus, Pavlos (from Arvanites), Tithorea, Chaironeia]. **Vúzë** (in Arvanitika). *Peloponnese*: Kyras Vrysi (from Arvanites). **Μπούζα (búza)**. *Central Greece*: villages of south Karpenissi, Lithochori and Kerasochori of Evrytania. **Βουζοβάτραχος (vuzovátrachos).** *Peloponnese:* Kiato of Corinthia. **Βαλτόβουζα** (**valtóvuza**). *Peloponnese*: Feneos Corinthias. **Μπακόβουζα (bakóvuza)**, *Peloponnese*: Ancient Feneos, Goura, Messino Corinthias.

**2.1.3. Μπζάκα (bžáka) and derivatives.**

**Μσάκα** **(mšáka)**. *Ionian Islands*: Lefkada. *Central Greece*: Agrinio, Arta (extremely common in all surrounding villages, e.g. Limini), Karpenissi. **Μισάκας (misákas)**. *Ionian Islands*: Lefkada. (almost everywhere, e.g. Agios Nikitas). **Μσιάκα (msiáka)**. *Central Greece*: Agrinio (Lepenou), Arachova, Ksiromero, common throughout Aetolia-Acarnania and Evrytania, Katouna, Mataranga, Milia, South Evrytania (Prousos, Tornos, Kastania), Stanos, Stranoma, all of Trichonida province. **Μψιάκα (mpsiáka)**. *Epirus*: city of Preveza. **Μουσάκα (musáka)**. *Ionian Islands*: Cephalonia; *Central Greece*: Nafpaktos, Potamoula. **Μπζάκα (****b****žáka) / Μπζιάκα (bziáka) / Μπσιάκα (bsiáka)**. *Epirus*: city of Preveza, Lakka Souliou. *Central Greece*: Amfilochia, Ambelakia, Areiada, Valtos province, Stylia, Loutro-Anoixiatiko, Neokastro, Famila, Palaiopyrgos, Parakampylia, Sardinia Amfilochias, Stathas Aitoloakarnanias, Arta, Western Doris (Pentagiou, Krokilio, Efpalio, Klima), Ftelia, Phocis, Kechrinia, Koboti, Malesiada, Pappadatos, Triklino, mountainous Nafpaktia, Chalkiopoulo. **Μπουσάκα (busáka)/ Μπουζάκα (buzáka)**. *Ionian Islands*: Cephalonia (everywhere), Corfou [1], Zakynthos (everywhere, e.g. Langadakia). **Μπουσάκλα (busákla).** *Peloponnese*: Ilia (Gastouni, Kolokitha, Loukavitsa, Markopoulo, Stafidokambos, Pineios dam and nearby villages, e.g. Roupakia, Velanidi, Agios Ilias), Vartholomio. **Βουσάκλα (vusákla)**. *Peloponnese*: Tzami Pineias. **Μπζάκλα (bžákla)**. *Central Greece*: Vitrinitsa-Tolofonas.

**2.1.4. Ζάμπα (žába) and derivatives.**

**Ζάμπα (žába*,* zába*,* or zámba).** *Epirus*: common primarily in Thesprotia, in the villages between Igoumenitsa and Ioannina, in all of the Filiatochoria, in the Eastern borders with Albania (Sagiada), in the Greek-speakers of Dropoli = Dropull [1], and in Preveza [area of Fanari (Arvanites), Themelo]. *Central Greece*: Desfina. *Thessaly*: Mega Monastiri of Larisa. *Thrace* (precise locality not known). *Ionian Islands*: Corfou (dominant zoonym on the island). *Macedonia***:** common in Western Macedonia, in Florina (among both Slavophones and Arvanites, e.g. in Lechovo), Pieria (Dion, among Greek-speakers and Vlachs), Imathia (Meliki) and Kastoria (Lagka). *Peloponnese*: Ilia [Akropotamia, Geraki, environs of Olympia, e.g. Achladini, Kallithea, Krestena, Strefi, Pyrgos, Tripiti [1], Chavari, Zacharo], Messinia (Siamou), Arcadia (Vachlia, Dimitra, Kontovazaina, and Western Gortynia more broadly, valley of Ladonas, Tripoli). **Ζάμπια (zámbia or zápia).** *Peloponnese*: Mesa and Exo Mani (everywhere, e.g. Areopoli, Marathea, Myrsini, Loukadika, Prosilio, Ochia, Tseria). **Ζάπα (záppa).** *Albania*: Agioi Saranta = Sarandë. *Epirus*: Ioannina, Granitsa, Rodotopi, Zitsa. *Macedonia*: Mandra Kilkis (Arvanites). **Ζιάπα (ziápa).** *Albania*: Dropoli = Dropull [1]. *Epirus*: Ano Pedina, Amfithea, Grammenochoria, Thesprotia, Filiates, Kastanonas, Katsikas, Ioannina, Granitsopoula and Polidoro Zitsas Ioanninon (Vlachs), Kalochori Zitsas, Konitsa, Margariti (Arvanites), Petsali, Plataria Thesprotias (Vlachs), Prosilio, Mastorochoria Konitsas, Serviana. *Macedonia*: Petrokerasa, Skalochori Kozanis. **Ζιάπκα (ziápka)**. *Macedonia*: Sochos, Ossa Langada, Dimitritsi, Serres. *Thrace*: Soufli [1]. **Ζιάπκου (****ziápku)**. *Thrace*: Lavara (also as ziápkus-ziaplákus), Soufli Evrou. **Ζιάμπα (ziámba)**. *Albania*: Dropoli = Dropull [1]*. Epirus:* Arta, Agios Spiridon Artas, Ioannina prefecture (Vourbiani, Despotiko, Iliorrachi Konitsas, Langada Konitsas, Melingoi Dodonis, Palaiochori, Perama, Zalongo, Zotiko), Lakka Souliou, Preveza (Louros [1]), Ano Ravenia, all of Pogoni, Zagori (Elafotopos, Tservari, Elafos, Papingo, Kato Pedina, Kleidonia, Frangades), Skorpiona, Zervochori Thesprotias. *Macedonia*: Archangelos Almopias (Meglenite Vlach village), Grevena [Georgitsa (Vlachs)], Kastoria (Germas, Kostarazi) [1], Smixi (Vlachs), Perivoli (Vlachs), Trikomo [1], Imathia (Fitia, Naousa), Kozani (Avgerinos, all of Voio province, Neapoli, Siatista), outskirts of Thessaloniki (Langadas [1], Vertisko, Lagyna, Nigrita [1]), Ritini and villages of Pieria mountains. *Thessaly*: Marathea, Palamas, Proastio (among Karagounides), Skepari in Kalambaka. *Romania*: Isvoarele (residents are of Thracian Greek origin). **Ζιάμπουρ (ziámbur)/ Ζιαμπούρ (ziambúr)**. *Macedonia*: Ossa Langada. **Ζιάμπου (ziámbu)**. *Thrace*: Rizia Evrou. **Ζιάμπος (ziámbos)**. *Thrace*: Xilagani. **Ζιαμπαρόκ (ziambarók or žabarók).** *Macedonia*: Karpi Paikou (by Meglenite Vlachs). **Ζάμπακος (zámbakos)**. *Peloponnese:* Trifyllia, Kopanaki [2], Zoni in Arkadia [2]. **Ζιάμπακας (ziámbakas) / Ζάμπακας (zámbakas)**. *Epirus*: Ioannina, Kleidonia. *Macedonia*: Grevena (Agios Georgios), Kastoria, Kozani [Vythos [1], Damaskinia (by Vlachs), Krimini [1], Xrisavgi [1]], Riakia Pierias. **Ζάμλιακας (zámliakas) / ζάμπλιακας (zámbliakas).** *Central Greece*: Agios Konstantinos in Fthiotis [2]. **Ζάπλιακας (zápliakas) / ζαπλιάκι (zapliáki)**. *Macedonia*: Mandra Kilkis (Arvanites from Bulgaria). **Ζαμπάκι (zambáki)**. *Thrace*: Asproneri in Didymoteicho, Metaxades. **Τζάμπακας (tzámbakas)**. *Macedonia*: Pieria [1]. **Τζάμπλιακας (tzámbliakas)**. *Thrace*: Didymoteicho. **Τζιάμπλακους (tziámblakus) /τζιάμπλακας (tziámblakas)**. *Thrace*: Evros (Kastanies, Keramos, Marasia, Paliouri, Sofiko, Spilaio). *Macedonia*: Neo Souli, Nigrita [1]. **Τσζιάμπλακους (tsziámblakus)**. *Thrace*: Isaakio. **Τζιάμπλακος (tziámblakos) / τζιαμπλιακός (tziambliakós) / τζιαπλιακός (tziapliakós)**. *Macedonia*: Nigrita. *Thrace*: Evros (Arzos, Pentalofo, Spilaio). **Τζιαμπλάκ (tziamblák).** *Thrace*: Didymoteicho [1], Evgeniko. **Τζιαμπλιάκι (tziambliáki)**. *Thrace*: Evros (Dilofo, Therapeio). **Τζιαμπλιάκα (tziambliáka)**. *Thrace*: Evros (Kastanies). **Τσιαμπλιάκα (tsiambliáka).** *Thrace*: Pentalofos. **Τζιάμνακους (tziámnakus)**. *Thrace:* Evros (Elia). **Τζιάμπνακους (tziámbnakus).** *Thrace:* Evros (Mikri Doxipara, Plati). **Τζιάπκους (tziápkus) / ζιάπκους (ziápkus) / χουμότζιαμπα (humótziamba).** *Thrace*: Soufli [1].

**2.1.5. In Pomak.**

**Ζάμπα** **(žába)** (Glafki, Pachni = Pashevik, Porta, Kotyli community = Kozluca, Emonio = Valkanova, Dimari = Demirdžik, Melivoia, Echinos = Shahin, Satres = Sinikovo). **Ζέμπα (žéba)** (Rodopi, Xanthi), Ano Thermes = Lydža. **Κόρα ζέμπα** **(kóra žéba).** Echinos = Shahin. **Ζάπα (zápa)**. Pachni = Pashevik. **Γουρζάμπα** **(ğuržába)**. Kentavros = Ketenlik. **Τζάμπλακας** (**tzámblakas**). Mega Dereio = Büyük Dervent.

**2.1.6. In Slavophones (“ντόπια”).**

**Ζάμπα (žába)** / **Ζαμπαρόκ (žabarók).** *Macedonia:* Griva Kilkis and neighbouring villages, common in all Slavophone areas of Greece. **Ζιαμπαρόκ (ziabarók).** *Macedonia*: villages of Ptolemaida (Lefkothea, Kardia). **Κράσταβα ζάμπα (krástava žába)**. *Macedonia*: Vevi Florinas (likely a recent influence from the official language of North Macedonia). **Ζαμπλιάκι (zambliáki)**. *Macedonia*: Liti Thessalonikis. **Τζιάπκα (tziápka)**. *Macedonia*: Petroussa Dramas.

**2.1.7. Μπάτζιακας (bátziakas)** **and derivatives.**

**Μποζιάκας (bóziákas)**. *Macedonia*: Arnaia in Chalkidiki. **Μπάτσιακας (bátsiakas)**. *Macedonia*: Metagitsi (among Greek-speakers and Vlachs), Nikiti in Chalkidiki. **Μπάτσακας (bátsakas)**. *Macedonia*: Darnakochoria (Neo Souli, Emmanouil Pappas, Pentapolis, Agio Pnevma, Chryso). **Μπατσιακομάνα (batsiakomána)**. *Macedonia*: all the Chasika villages – Mantemochoria. **Μπάτζιακας (bátziakas)**. *Macedonia*: Chalkidiki [1]. *Thrace*: Evros (Ambelakia, Ellinochori, Karoti, Mani, Ormenio, Patagi, Pyrgos of Orestiada, Didymoteicho [1], Ftelia, Fylakio, Sitochori, Sterna, Chionades). **Μπατζιακός (batziakós).** *Macedonia*: Nea Vyssa Orestiadas (also used by the Arvanite residents). **Βατραχομπατσιακός (vatrachobatsiakós)**. *Thessaly*: Tyrnavos, **Μπαμπάτσκους μπάτζιακας (babátskus bátziakas).** *Thrace*: villages of Orestiada. **Μπατζακός (batzakós)**. *Thrace*: Evros (Dikaia). **Μπατζάκια (batzákia).** *Thrace*: Orestiada**. Μπατζιάκι (batziáki).** *Thrace*: Orestiada (Lepti). **Μπατζακούδ’ (batzakúd).** *Thrace*: Evros (Koufovouno). **Μπατζαρόκα (batzaróka)**. *Macedonia*: Agios Vasileios of Thessaloniki (by refugees).

**2.1.7. Μπράσκα (brásca) and derivatives.**

**Μπράσκα (brásca)**. *Epirus*: one of the most common toad zoonyms in Epirus, Agrinio, and Arta (Megalochari [1]), Valanidoussa Prevezas, Ioannina, Milia and Chrysovitsa in Metsovo, Kryovrisi Ioanninon, Palaiochori Syrrakou (Vlach village), Flambourari (Vlach village), Zagori [Asprangeloi, Anthrakitis, Vrisochori (Vlach village), Vovousa (Vlach village), Vitsa, Dilofo, Dipotamo, Elatochori, Kavallari, Leptokaria, Kipoi, Makrino, Miliotades, Monodendri, Negades, Petra, Tsepelovo]. *Thessaly*: dominant toad zoonym in Thessaly (both on the plains and the mountains); Aspropotamos (Polythea, among Vlachs), Vatsounia, Gomfoi, Grizano, in most Karagoun villages [Astritsa (as *μπράχτσα*), Agios Prokopios Trikalon, Anthiro Karoplesiou, Vlochos (*μπράτσα*), Dendrochori Trikalon (*μπράχτσα*), Ermitsi (*μπράχτσα*), Gorgovites, Itea (*μπράχτσα*), Kaminades Karditsas, Keramidi (*μπράχτσα*), Krini Farsalon, Fyllo (*μπράχτσα*), Magoula, Magoulitsa, Makrichori, Mascholouri, Mataranga Karditsas (*μπράχτσα*), Megala Kalyvia, Melissochori Sofadon, Mizdani (Agnantero), Myrini, Kallifoni, Karditsomagoula (with *μπράχτσα* considered as the original Karagoun accent), Koskina, Kranea (*μπράχτσα*), Kypseli, Agioi Anargyroi (Paliouri Goritsas), Sitochoro, Sikeona, Palaioklissi, Palamas, Paschalitsa, Proastio, Frangos, Filia Karditsas, Rizovouni, Stavros, Nomi, Trikala], Livadi in Olympus (Vlachs), Mesochora, Karditsa, Fanari and Prodromos Karditsas, Kastania Kalambakas (Vlachs), Elassona, Kalithea Kalambakas, Keramidi, Klokotos, Larisa, Megala Kalyvia, Nea Anchialos, Pelion (in all villages, e.g. Anilio, Ano Lechonia, Agios Georgios Nileias, Kissos, Mouresi, Nileia, Neochori, Perivlepto, Tsangarada), Pelasgia-villages of Othrys, Pyli, Stournaraiika , Drosochori (Tifloselli), Trikala, Tirnavos. *Thrace*: Loutra Evrou (among Sarakatsani), Polisitos. *Macedonia*: Palaios Panteleimonas in Dion, Grevena, Prionia Grevenon (Vlachs), Zarkadia in Kavala (among Sarakatsani). *Peloponnese*: Argolis (Dimaina, Vivari, Karyes, Krya Vrysi, Platani, Lefkakia in Nafplion, Ligourio, Skafidaki, Skoteini), Corinthia (Riza), Arcadia [Asea, Kollines, Vervena [1], Stolos, Tegea, Tripolo, Tsakonia (Prastos), Nestani, Kandila, Lykochia, Agios Nikolaos, Mantineia, Kastri and Kosmas in Kynouria, Levidi, Megalopoli], commonest toad zoonym in Messinia, Laiika Messinias, Longa, Trifilia-Olympia, Gargalianoi [1], Bardounochoria (Xirokambi), all of non-Maniot Laconia [e.g. Agios Dimitrios in Zarakas, Anogeia, Vresthena [1], Agios Konstantinos [1], Longanikos, Kastoreio, Kastri, Karya, Koniditsa, Pellana, Skoura, Sparti), Skala, Monemvasia and surrounding villages]. *Central Greece*: Pockets in Aetolia-Acarnania (e.g. Katouna, Messolonghi, Bouka Amfilochias, Chrysovitsa in Xiromero), Agrinio, Agios Dimitrios in Nafpaktos, Achinos, Domianoi, Ambelakiotissa Nafpaktias, Arachova Nafpaktias, Trani Lakka Nafpaktias, Atalanti, Attica, especially in Parnitha (among Sarakatsani), Glyfa, North Evrytania [commonest zoonym, e.g. Agrafa, Trovato, villages of Karpenissi (e.g. Diplatano)], Lamia, Loukissia Chalkidas (among Sarakatsani), Euboea (Agia Anna, Vassilika, Vlachia, Istiaia, Kampia, Limni, Lichada, Manikia, Mantoudi, Metochi, Neos Pyrgos, Pano Steni, Papades, Pefki, Pili, Prokopi), Doris (Artotina), Domnista, Perkos [1], Amfikleia, Dadi, Tithorea, Domokos, Polydrosos [1], Spercheiada, Ypati [1], very common in northern mountainous Phocis (e.g. Chrisso, Kastriotissa, Mavrolithari). *North Aegean*: Samos (Lekka [1], Palaiokastro, Pagondas, Chora). **Μπρασκούλα (brascúla)**. *Macedonia*: Agia Paraskevi in Chalkidiki. **Μπρασκακούλα (brascacúla)**. *Central Greece*: Euboea (Katounia Limnis, Skepasti). **Μπρασκ’ (brásc’)**. *Thessaly*: Kokkinopilos Elassonas (Vlach village). **Μπράσκια (bráscia)**. *Epirus* (Vitsa Ioanninon, Kapesovo). **Μπράσκλα (bráscla)**. *Epirus:* Piges Artas. *Thessaly*: Argithea, Trikala, Pyli Trikalon, Karditsa [1], Drakotrypa. *Central Greece*: Vitrinitsa. *Peloponnese*: Vresthena. *Central Greece*: Agrafa. **Μπρόσκα (brósca)** (only used by Vlachs). *Albania*: Selenicë in Vlorë. *Macedonia*: Archangelos in Almopia (by Moglenite Vlachs), Pella, Rodolivos in Serres. **Μπρόσκο (brósco)** (only used by Vlachs). *Macedonia*: Prosotsani. **Μπρόσκου (bróscu)** (only used by Vlachs). *Macedonia*: Asvestochori in Thessaloniki, Esovalta in Giannitsa, Livadia in Mt Paiko (Gramousian Vlachs), Polykastro Kilkis, Kilkis. *Epirus*: Aetomilitsa, Metsovo. *Thessaly*: Almyros. **Μπρουόσκα (bru****ósca)** (only used by Vlachs). *Macedonia*: Paiko. **Μπρουάσκα (bruásca)** (only used by Vlachs). *Bulgaria*: Iagodovo, Plovdiv. *Epirus*: Anilio in Metsovo, Distrato (Vlach village), Milia in Metsovo. **Μρουάσκα (mruásca)** (only used by Vlachs). *Epirus*: Laista (Vlach village). **Μπρουάτικου (bruáticu) /Μπροάτικου (broáticu)** (only used by Vlachs). *Thessaly*: Falani, Chaliki in Aspropotamos (Vlach village), Cheimadi in Larisa, Kalyvia in Elassona, Koutsoufliani in Kalambaka (Vlach village), Chrysomilia in Kalambaka, area of Katara (Arvanitovlachs). *Macedonia*: Dion (only among the Vlach residents), Xirolivado Veroias (Vlach village), Karitsa in Pieria (Vlach village), Serres (among Vlach residents), Seli in Veroia (Vlach village), Diavatos in Veroia. **Μπράτικου (bráticu)** (only used by Vlachs). *Epirus*: Paleoselli on Mt Smolikas (Vlach village). *Macedonia*: Samarina in Grevena (Vlach village). *Thessaly*: Livadi on Mt Olympus (Vlach village), Tziourtzia in Trikala (Vlach village). **Μπριάσκα (briásca)**. *Thessaly*: Aidona (Vlach village), broader area of Aspropotamos in Trikala (Vlach villages), Glikomilia in Kalambaka, Marko in Karditsa (among Karagounides), Larisa [1]. *Macedonia*: Vrontou on Mt Olympus. **Μπουράσκα (burásca).** *Peloponnese*: Argolis [Adami (possibly had speakers of Arvanitika in the past), Koliaki, Tracheia]. **Μπαρδαμπράσκα (bardabrásca) /μπαρδαμπάσκα (bardabásca)**. *North Aegean*: Samos.

**2.1.8. Θιθιλιώπα (thithiliópa) and derivatives.**

**Θιθιλιώπα (thithiliópa)**. *Epirus*: Karteri in Thesprotia (Arvanites). **Σισελιώπα (siseliópa)**. *Epirus*: Kastri in Thesprotia (Arvanites). **Τιθλιώπα (tithliópa) / Μπιθλιώπα (bithliópa)**. *Epirus*: Agia in Parga, Agia Triada-Bouratsa in Ioannina, Elefhterio in Thesprotia, Skiadas in Preveza, Tzoumerka (Kalarrytes, Syrrako, among Greek speakers). *Central Greece*: Euboea (Argyros) (only among Arvanites). **Χλιώπα (hliópa)**. *Epirus*: Terovo, Sklivani*,* Pesta (among Greek speakers), villages around Preveza (e.g. Filippiada) **Θυθλιώπα (thithliópa) /Θλιώπα (thliópa)**. *Central Greece*: Euboea (all Arvanite villages from Krieza to Steira and above, centred around Zarakes, Dramesi, and Kallianos).

**2.1.9. Μπρέσκλα (bréskla) and derivatives.**

**Μπρέσκλα (bréskla).** *Epirus*: Arta and environs (e.g. Skoupa), Konitsa, mountain villages of Ioannina (e.g. Platanoussa, Tzoumerka), environs of Preveza (e.g. Agios Georgios). **Μπρέσκα (bréska)**. *Epirus*: Mouzakaioi, Amfithea. *Central Greece*: Euboea (among Arvanites in Amygdalia of Kavodoro, and Karystos, and among Greek-speakers in Platanistos), Neochoraki Thivas (Arvanites).

**2.2.1. Μπερτικοσα (bertikósa) and derivatives.**

**Μπερτικοσα (bertikósa)**. *Thrace*: Paradimi in Komotini (among Arvanites from East Thrace). *Peloponnese*: Argolis (Angelokastro, Dimaina, Methana). *Central Greece*: Aspropyrgos, Avlona, Vari, Varnavas, Grammatiko, Kalamos, Koropi, Kriekouki, Magoula, Chasia in Attica, Boeotia [Vagia, Vathi, all Dervenochoria (e.g. Prasino, Skourta), Domvraina, Thisvi, Kleidi, Kyriaki, Leontari of Thiva, Plataies], Euboea [all places where Arvanitika is spoken, e.g. Almyropotamos, Gavalas (also known as *φουρνία*), Krieza, Mesochoria in Carystia, Kalentzi (formerly Greek-speaking), Katsaroni], Malesina. *Cyclades*: Andros (Arni, Katakoilos, Aprovatou). **Περτικόσε (pertikóse)**. *Central Greece*: Attica (Spata). **Πρετικός (pretikós).** *Central Greece*: Zeriki**. Μπρτσκ (brtsk) / Μπρτσκα (brtska)**. *Thrace*: Rigio-Cheimonio in Evros. *Central Greece*: Dervenochoria (referring to the tortoise). **Μπρτόσκα (brtóska)**. *Thrace*: Tychero in Evros.

**2.2.2. Μπρασκαφούτα (brascafúta) and derivatives.**

**Μπρασκαφούτα (brascafúta).** *Epirus*: Aetopetra (Kossoliani) of Ioannina. *Central Greece*: Euboea (Agriovotano). *Peloponnese*: Messinia (Kouvelas). **Μπασκαφούτα (bascafúta).** *Peloponnese*. Messinia (Avlona in Trifilia, Trifilia, Ripesi-Kevalovrysi, Kopanaki, Kyparissia, Pylos, Siamo, Filiatra). **Ασκουφούτα (ascufúta)**: *Peloponnese*: Arkadia (Stolos).

**2.2.4. Κούβακας (kúvakas) and derivatives.**

**Κούβακας (kúvakas)** (plural *κουβάκοι*). *North Aegean:* Chios (Agios Georgios Sykousis, Armolia, Avgonyma, Vasileonoiko, Vrontados, Volissos, Didymes, Tholopotami, Thymiana, Kalamoti, Kardamyla, Katarraktis, Koini, Livadia, Lithi, Mesta, Neromyloi, Nechori, Olympoi, Pispilounta, Spartounta, Sikiada, Flatsia, Zifias, Chora). *Turkey*: Çeşme. **Κουβακομάνα (kuvakom****ána)**. Afrodisia. **Κούγακας (kúğakas)**. Viki, Keramos. **Κούακας (kúakas)**. Pyrgi, Pytios, Fytousiki-Kipouriani.

**2.2.5. Φαρφαλιώπα (farfaliópa) and derivatives.**

**Φαρφαλιώπα (farfaliópa)**. *Epirus*: Karvounari, Plataria, Zitsa, Geroplatanos in Pogoni (Arvanitovlachs), Paramythia, Margariti (Arvanitovlachs), Dendrochori in Kastoria. *Macedonia*: Krya Vrysi in Pella (Arvanitovlachs), Vrontero in Florina. *Thessaly*: Almyros. **Φαρφαλιώπε (farfaliópe).** *Epirus*: Kefalovryso in Pogoni, Vissani in Ioannina (Arvanitovlachs). **Φιρφιλιώπα (firfiliópa).** *North Macedonia*: Nižepole. *Thessaly*: Nea Zoi, Sesklo (Arvanitovlachs). **Χαρχαλιώπα (harhaliópa)**. *Central Greece*: Palaiomanina and Stratos of Aetolia-Acarnania (by residents formerly known as Arvanitovlachs, now as Karagounides, unrelated to the homonymous population of Thessaly). **Θαρθαλούπα (tharthalúpa)**. Syrrako of Ioannina (Vlachs). **Θουρθουλούπα (thurthulúpa)**. Tzoumerka (Syrrako, Kalarrytes, Matsouki) (Vlachs).

**2.2.6. Σταμπόλ (stamból), στάμπολ (stámbol).**

**Σταμπόλ (stamból), στάμπολ (st****ámbol).** *Thrace:* Myki = Mustaftsova, Sminthi, Prosilio = Pulevo, Alma = Egnila, Mantaina = Basaykovo, Siroko = Shiroka, Gorgona = Bratankova, Eora = Ljulka).

**3. References**

[1]. Lithoxou D. (2019) Romaiika and Greek. The language is voices, not letters. Dimitris Lithoxou: Nov 4 2019 [cited 2022 Feb 13]. In: [www.lithoksou.net](http://www.lithoksou.net) [Internet]. Greece: 2006- [70 screens]. Available from: <https://www.lithoksou.net/2020/11/romaiika-kai-ellinika.html> [accessed on 13/02/2022]. [in Greek]

[2] Unpublished Archive of the Research Centre of Modern Greek dialects and Idioms-Historical Dictionary of modern Greek of the Academy of Athens. Athens, Academy of Athens, manuscripts 392, 8; 858, 35. [accessed on 10/02/2023]
